# Assessment of the exchange-hole dipole moment dispersion correction for the energy ranking stage of the seventh crystal structure prediction blind test

**DOI:** 10.1107/S2052520624002774

**Published:** 2024-10-15

**Authors:** R. Alex Mayo, Alastair J. A. Price, Alberto Otero-de-la-Roza, Erin R. Johnson

**Affiliations:** a Department of Chemistry Dalhousie University 6243 Alumni Crescent Halifax Nova Scotia B3H 4R2 Canada; b Departamento de Química Física y Analítica and MALTA-Consolider Team, Facultad de Química Universidad de Oviedo 33006Oviedo Spain; CSIR–National Chemical Laboratory, India

**Keywords:** crystal structure prediction, density-functional theory, London dispersion, free-energy correction

## Abstract

The seventh blind test of crystal structure prediction (CSP) methods substantially increased the level of complexity of the target compounds relative to the previous tests organized by the Cambridge Crystallographic Data Centre. In this work, the performance of density-functional methods is assessed using numerical atomic orbitals and the exchange-hole dipole moment dispersion correction (XDM) for the energy-ranking phase of the seventh blind test. Overall, excellent performance was seen for the two rigid molecules (XXVII, XXVIII) and for the organic salt (XXXIII). However, for the agrochemical (XXXI) and pharmaceutical (XXXII) targets, the experimental polymorphs were ranked fairly high in energy amongst the provided candidate structures and inclusion of thermal free-energy corrections from the lattice vibrations was found to be essential for compound XXXI. Based on these results, it is proposed that the importance of vibrational free-energy corrections increases with the number of rotatable bonds.

## Abstract 2

Density-functional calculations using numerical atomic orbitals and the exchange-hole dipole moment dispersion model were applied to the energy ranking stage of the seventh crystal structure prediction blind test. This methodology gave successful predictions of the experimentally isolated polymorphs as being low in energy for three of the compounds considered, and for one additional compound upon inclusion of a vibrational free-energy correction.

## 1. Introduction

First-principles molecular crystal structure prediction (CSP) is a grand challenge in the field of computational chemistry and is fruitful ground for ongoing method development. To assess the performance of various methods for both the structure generation and energy ranking stages of CSP, the Cambridge Crystallographic Data Centre (CCDC) regularly holds blind test competitions. Solved but unpublished crystal structures for a small set of compounds are held in reserve by the CCDC and participants have a set period of time to submit predicted crystal structures given only chemical diagrams of the compounds. There have been six previous blind tests to date (Lommerse *et al.*, 2000 ▸; Motherwell *et al.*, 2002 ▸; Day *et al.*, 2005 ▸, 2009 ▸; Bardwell *et al.*, 2011 ▸; Reilly *et al.*, 2016 ▸), with the seventh held between 2020 and 2022 (Hunnisett *et al.*, 2024*a* ▸; Hunnisett *et al.*, 2024*b* ▸).

In the fourth (Day *et al.*, 2009 ▸), fifth (Bardwell *et al.*, 2011 ▸), and sixth (Reilly *et al.*, 2016 ▸) blind tests, dispersion-corrected density-functional theory (DFT) was shown to provide large improvements in energy ranking of candidate crystal structures compared to classical force-field methods. The Neumann group was the first to use DFT in the blind tests with their own empirical dispersion correction (Neumann & Perrin, 2005 ▸; Neumann *et al.*, 2008 ▸; Kendrick *et al.*, 2011 ▸) and applied their methodology to the earlier blind tests retroactively (Asmadi *et al.*, 2009 ▸). The more popular D3 (Grimme *et al.*, 2010 ▸, 2011 ▸) and MBD (Tkatchenko *et al.*, 2012 ▸; Ambrosetti *et al.*, 2014 ▸) dispersion corrections have been used by other participants in submissions to the subsequent blind tests and also showed good performance (Brandenburg & Grimme, 2016 ▸; Reilly *et al.*, 2016 ▸; Hunnisett *et al.*, 2024*a* ▸).

Dispersion corrections are routinely paired with generalized gradient approximation (GGA) functionals when modelling molecular crystals. However, GGAs suffer from delocalization error (Cohen *et al.*, 2008*b* ▸; Kim *et al.*, 2013 ▸; Bryenton *et al.*, 2023 ▸), which can lead to overstabilization of organic salts, halogen bonds, cooperative hydrogen-bonding networks, and extended π conjugation. Delocalization error can be reduced through subsequent single-point energy evaluations using hybrid functionals, for example with PBE0-MBD, as has been used in the sixth blind test and several other CSP studies (Marom *et al.*, 2013 ▸; Shtukenberg *et al.*, 2017 ▸; Hoja & Tkatchenko, 2018 ▸; Hoja *et al.*, 2019 ▸; Mortazavi *et al.*, 2019 ▸). Quite recently, we assessed the performance of our exchange-hole dipole moment (XDM) dispersion correction (Johnson, 2017 ▸; Price *et al.*, 2023*b* ▸) in combination with hybrid functionals for the compounds appearing in all six previous blind tests (Price *et al.*, 2023*a* ▸). While delocalization error adversely affects the energy ranking provided with GGA functionals for organic salts in the fifth and sixth CSP blind tests (Whittleton *et al.*, 2017*a* ▸), and leads to errors in the intramolecular conformational energies of many conjugated molecules (Whittleton *et al.*, 2017*b* ▸; Greenwell & Beran, 2020 ▸; Greenwell *et al.*, 2020 ▸; Beran *et al.*, 2022 ▸), XDM-corrected hybrid functionals were shown to yield improved performance.

One of the key outcomes from discussion surrounding the sixth blind test was the idea of surpassing ‘zeroth-order’ CSP (Price, 2018 ▸), using only the electronic energies, by inclusion of thermal free-energy corrections (Hoja *et al.*, 2017 ▸). Such corrections have previously been used to convert experimental sublimation enthalpies to lattice energies in development of the C21 and X23 benchmarks of small-molecule crystals (Otero-de-la-Roza & Johnson, 2012 ▸; Reilly & Tkatchenko, 2013 ▸; Dolgonos *et al.*, 2019 ▸), as well as for studies of aspirin polytypes (Reilly & Tkatchenko, 2014 ▸). However, the importance of these corrections for CSP has been a matter of some debate in the literature, with (Nyman & Day, 2015 ▸) showing they are generally small – less than 2 kJ mol^−1^ in 94% of cases taken from a set of 508 polymorphic compounds – using a distributed multipole force field. For a set of 17 polymorph pairs, DFT calculations confirmed these corrections to be ∼ 2 kJ mol^−1^ or less (Weatherby *et al.*, 2022 ▸), although this study was limited to small, rigid molecules. DFT thermal free-energy corrections were also found to be of comparable magnitude for polymorphs of coumarin (Shtukenberg *et al.*, 2017 ▸) and rotigotine (Mortazavi *et al.*, 2019 ▸). Conversely, analogous calculations on the compounds from the sixth blind test occasionally showed significantly larger thermal free-energy corrections, up to 8.4 kJ mol^−1^ for the highly flexible compound XXIII (2-{[4-(3,4-dichloro­phenethyl)phenyl]amino}benzoic acid; Hoja & Tkatchenko, 2018 ▸; Hoja *et al.*, 2019 ▸).

**Figure 1:**
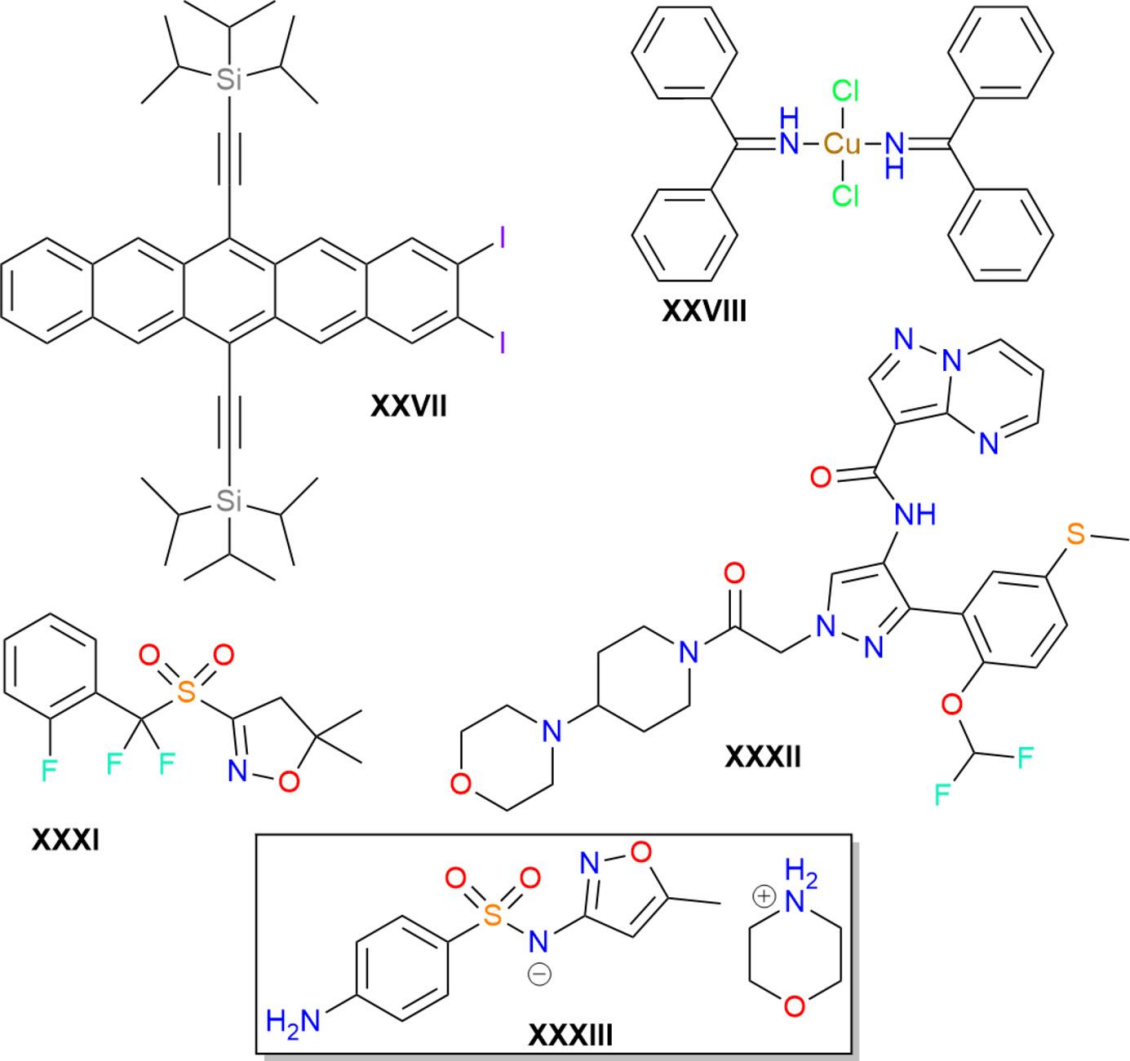
The five compounds considered for the energy-ranking phase of the seventh CSP blind test. XXXIII is an organic salt, with both ions shown within the box present in a 1:1 stoichiometric ratio.

The seventh CSP blind test was separated into two phases, with the first focusing on structure generation and the second on energy ranking. In this second phase, sets of candidate crystal structures were provided by the CCDC for the five compounds shown in Fig. 1 ▸. These sets comprised 100 structures each for compounds XXVII and XXXI, and 500 structures each for compounds XXVIII, XXXII, and XXXIII. Within each set, one or more seed structure(s) (see Table 1 ▸) were provided that corresponded to the experimental crystal structure(s) after geometry optimization with a CSD-tailored force field (Hunnisett *et al.*, 2024*a* ▸). For compound XXVII, two equivalent instances (see below) of the low-temperature experimental form were included as seed structures (rotation of the isopropyl groups leads to a disordered form at high temperature). For the copper compound, XXVIII, only one polymorph was isolated experimentally and included as a seed structure. For the agrochemical compound XXXI, the provided set contained four experimental seed structures. One of these seeds corresponds to form B, which was found to be the most stable polymorph experimentally at low temperatures. At high temperature, a disordered form A became more stable; both the major and minor components of this form were included as seed structures. The final experimental seed structure for XXXI corresponds to the desolvate form C. For the large, flexible, drug-like compound XXXII, two experimental seed structures were provided, corresponding to the more stable form B and the major component of the less stable, disordered form A. Lastly, for the organic salt, XXXIII, two experimental seed structures were provided, with form B again more stable than form A, which was found to be a disappearing polymorph.

The present work reports on the performance of several XDM-corrected density functionals for the energy ranking phase of the seventh CSP blind test. Within this phase, our group was one of ten that employed dispersion-corrected DFT, with four of these using hybrid functionals; four groups also included thermal free-energy corrections (Hunnisett *et al.*, 2024*a* ▸). Due to time constraints, we were not able to include free-energy corrections in our blind test submission, but do include them here for selected low-energy structures of compounds XXXI and XXXII. In this article, we will focus only on presentation of our own group’s results. Interested readers are directed to Hunnisett *et al.* (2024*a* ▸) for a comparison of the results across groups. Our methodology was able to rank the most stable experimentally isolated polymorph as the lowest in energy for two of the five compounds considered (XXVIII and XXXIII), and second lowest for one additional compound (XXVII). However, for the other two compounds (XXXI and XXXII) the isolated polymorphs were ranked substantially higher energetically. For compound XXXI, this result is unsurprising for form C since it possesses large crystal voids, while form B is correctly predicted to be the most stable polymorph upon inclusion of thermal free-energy corrections arising from the lattice vibrations. Including thermal free-energy corrections also yields an improved ranking for compound XXXII, and we conjecture that thermal and kinetic effects become increasingly important as the number of rotatable bonds increases.

## 2. Computational methods

### 2.1. Crystal structure comparison

A key concern in assessing CSP methods is how to quantify whether a candidate structure is the same or different when compared to the experimental polymorph. By far the most common method for crystal structure comparison is the COMPACK algorithm (Motherwell & Chisholm, 2005 ▸), implemented in the CCDC’s *Mercury* program (Macrae *et al.*, 2020 ▸). COMPACK matches molecules within a given cluster size (commonly 20) by generating an optimal overlay that minimizes the root-mean-square deviation (RMSD) in the atomic positions. However, we have recently illustrated a problem with COMPACK that precludes its routine application to molecules with several highly branched functional groups (Mayo *et al.*, 2022 ▸), such as compound XXVII in the seventh blind test. This problem likely arises from the application of a graph-matching algorithm (Ullmann, 1976 ▸) to identify matching molecules within the structures and align them.

COMPACK can also be quite sensitive to choices of cutoff values (Mayo & Johnson, 2021 ▸; Mayo *et al.*, 2022 ▸) and cluster sizes (Mayo *et al.*, 2022 ▸) that make its common use as a black-box method concerning. As a result, we have argued that distance-based methods like COMPACK should be used in conjunction with comparison methods based on powder X-ray diffraction (PXRD) to provide more certainty in the comparison results than can be achieved with either type of method alone.

PXRD-based methods quantify structure similarity using the overlap of the powder diffractograms of a pair of crystal structures. Among other ways, the overlaps can be computed using de Gelder’s cross-correlation function (de Gelder *et al.*, 2001 ▸). The dissimilarity metric is then the powder pattern difference (PWDF), defined as one minus the overlap, such that exactly matching structures have a PWDF score of zero. However, PXRD-based comparison methods are only effective for pairs of crystal structures obtained under the same experimental conditions, or optimized with the same level of theory for *in silico* generated structures. This is because the PXRD peak locations are highly sensitive to the unit-cell dimensions, which change depending on temperature, pressure, and the particular choice of computational method. To overcome this limitation, we developed the variable-cell powder difference (VC-PWDF) method (Mayo *et al.*, 2022 ▸). VC-PWDF works by exploring a range of possible unit-cell definitions and distorting the cell vectors before evaluating the powder pattern difference. Overall, VC-PWDF was shown to agree with COMPACK in over 97% of almost 45 000 individual crystal-structure comparisons in the CSD (Mayo *et al.*, 2022 ▸), although it can provide incorrect classification of polytypes and conformational phases because of the similarity in their unit cells.

In this work, we employ a mix of RMSD(20), PWDF, and VC-PWDF methods for structure comparison. The RMSD(20) values were obtained using the Python API interface to *Mercury* (Macrae *et al.*, 2020 ▸), while PWDF and VC-PWDF scores were obtained using the *critic2* program (Otero-de-la-Roza *et al.*, 2014 ▸).

### 2.2. DFT calculations

The geometries of all provided candidate crystal structures were fully optimized using an in-house modified version of the *FHI-aims* program (version 210513; Blum *et al.*, 2009 ▸). Geometry optimizations were performed using the B86bPBE functional (Becke, 1986 ▸; Perdew *et al.*, 1996 ▸) with the XDM dispersion correction (Price *et al.*, 2023*b* ▸), with the ‘lightdenser’ basis set and integration grid, and a relaxation convergence threshold of 0.005 eV Å^−1^. Subsequent single-point energy calculations were then performed on all optimized structures using either the hybrid B86bPBE-25X or B86bPBE-50X functionals with the same lightdenser basis set and grid settings, as well as with the B86bPBE functional with the tight basis settings, all with XDM dispersion. Price *et al.* (2023*b* ▸) give the relevant damping parameters. Based on previous tests (Blum *et al.*, 2009 ▸), and excellent agreement with planewave results (Lejaeghere *et al.*, 2016 ▸; Price *et al.*, 2023*b* ▸), there should be negligible basis-set superposition error. For compound XXVIII, all calculations were run for a ferromagnetic configuration with one unpaired electron per copper atom. The single unpaired *d* electron is evident since Cu^II^ will have an electronic configuration of [Ar]3*d*^9^, and a ferromagnetic configuration is required in order to have a proper spin eigenstate that can be well described by Kohn–Sham DFT (Cohen *et al.*, 2008*a* ▸).

**Figure 2:**
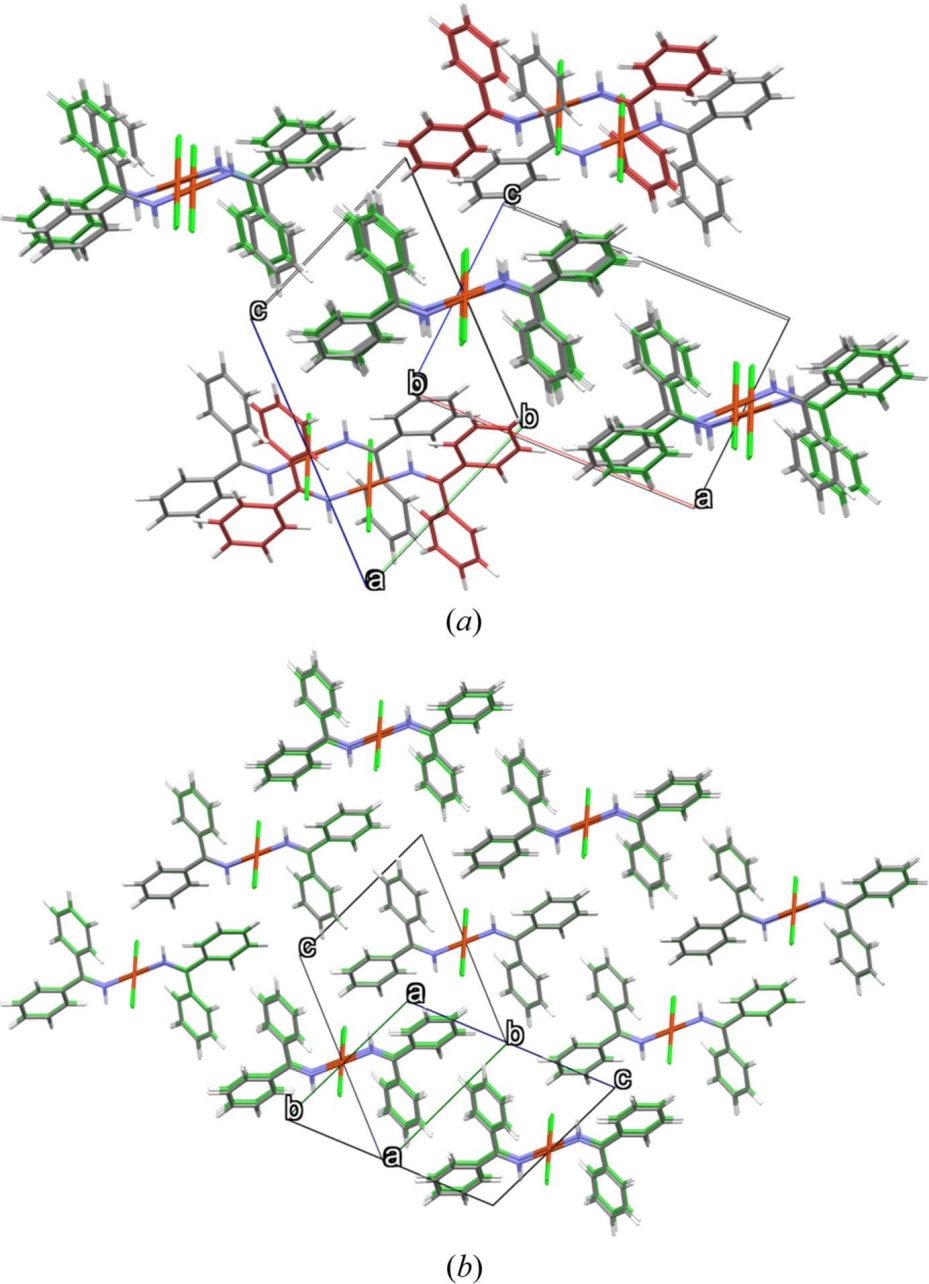
COMPACK overlays of structure 145 of compound XXVIII and the reference experimental polymorph. Results are shown for the initial structure provided by the CCDC (top) and after full geometry optimization (bottom) with a convergence threshold of 0.005 eV Å^−1^.

We note that there are differences in the relative energies and computational timings between this work and our blind test submission, due to the time constraints for the blind test and the fact that we were still making changes to the code when its second phase began. In this work, all geometry relaxations started from the crystallographic information files (CIF) provided by the CCDC with the finalized XDM implementation reported by Price *et al.* (2023*b* ▸) and relaxed with a convergence threshold of 0.005 eV Å^−1^. However, in our blind test submission, we had relaxed all structures with a much looser threshold of 0.025 eV Å^−1^, and only completed relaxations with the 0.005 eV Å^−1^ threshold for candidates lying within 1.5 kcal mol^−1^ (6.28 kJ mol^−1^) of the minimum obtained with the looser convergence criterion. For the most part, the tighter convergence threshold did not substantially alter the relative energies. However, it did result in large geometry and energy changes for a handful of structures. One key example is structures 145 and 207 for compound XXVIII. Here, the initial structures differed from the experimental polymorph by a translation of some of the molecules in the unit cell (see Fig. 2 ▸) and they were (effectively degenerate) high-energy structures, lying > 30 kJ mol^−1^ above the minimum in our blind test submission. However, after full optimization with the tighter convergence threshold, the two structures became duplicate matches to the experimental form. Presumably, with the looser criteria, the optimization was deemed converged when the structure was occupying some nearly flat shoulder of a high-dimensional potential energy surface. With tighter criteria, the optimization continued and proceeded to follow a path with small negative gradients until it could move away from the flat shoulder and find a much more stable local minimum. This should serve as a warning to ensure sufficiently tight convergence criteria during geometry optimizations for CSP, despite the appeal of using looser criteria to reduce computational cost.

### 2.3. Harmonic vibrational calculations

To apply thermal free-energy corrections in the harmonic approximation, vibrational frequency (phonon) calculations were performed for the 16 lowest-energy candidates of compound XXXI and the 26 lowest-energy candidates of compound XXXII, according to the GGA/light ranking. These numbers correspond to the ranks of the least stable of the experimental seed structures with the GGA/light level of theory (excluding the high-energy solvate for compound XXXI). The phonon calculations used the finite-difference approach as implemented in the *phonopy* package (Togo & Tanaka, 2015 ▸). The energies for all supercells were again evaluated with B86bPBE-XDM, lightdenser setting, using *FHI-aims* (Blum *et al.*, 2009 ▸). For compound XXXI, the thermal free-energy corrections were converged to within 0.5 kJ mol^−1^ per molecule with respect to supercell size. For compound XXXII, the thermal free-energy corrections were only converged to within 1.0 kJ mol^−1^ per molecule due to the large unit-cell sizes, which made calculations on larger supercells intractable. Lists of the candidate structures carried forward to phonon calculations, and the corresponding supercells used, are given in the supporting information.

## 3. Results and discussion

### 3.1. Structure changes

**Figure 3:**
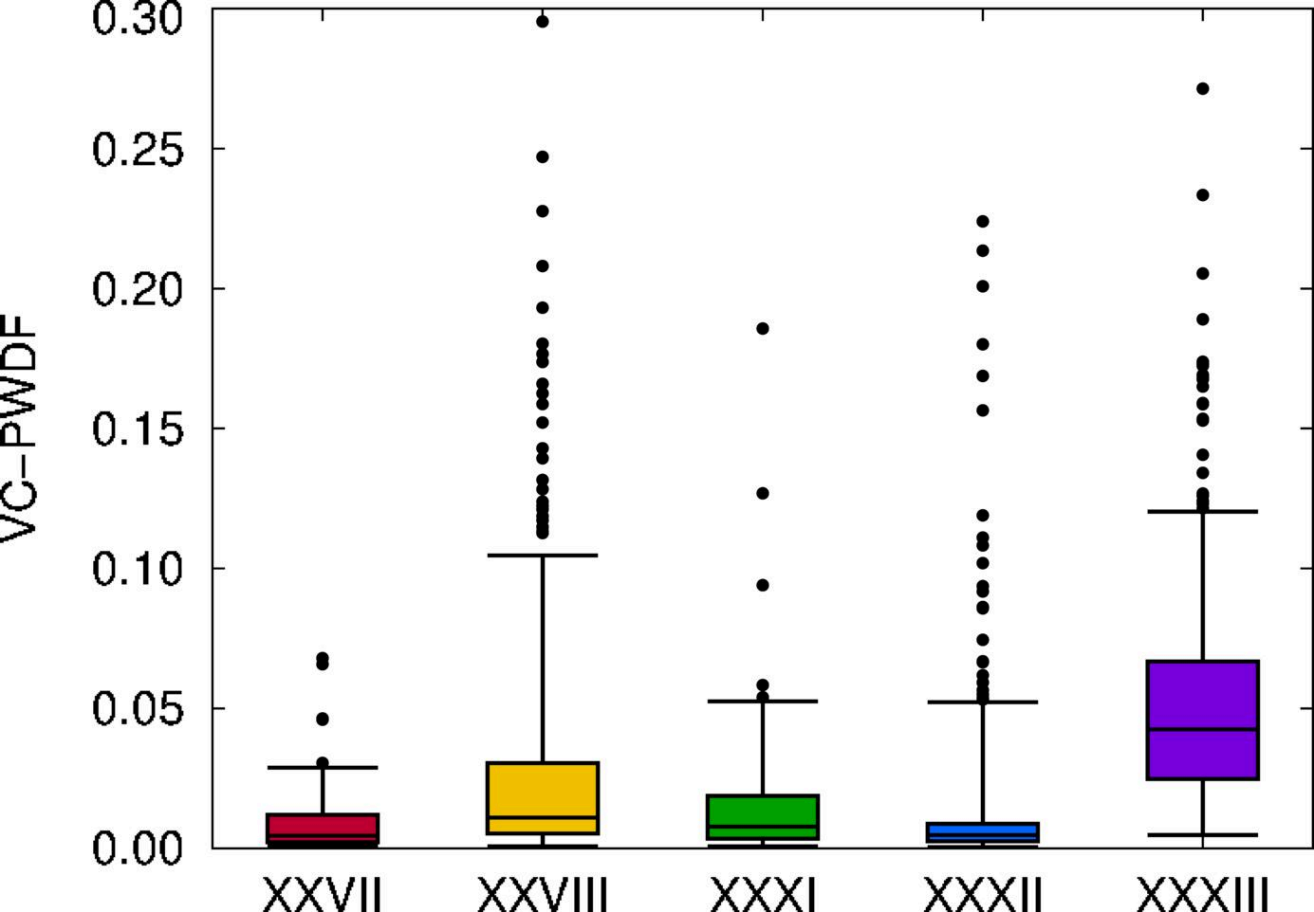
Box and whisker plot showing the extent of structural change resulting from DFT geometry optimization, as quantified by the VC-PWDF score between the provided and optimized structures for each candidate. The boxes show the interquartile ranges, the whiskers encompass 95% of the data, and remaining outliers are shown as individual points.

Because the candidate crystal structures provided by the CCDC were all optimized with a CSD-tailored force field, they may not correspond to stable minima with DFT. To assess how much structural change occurred during the DFT geometry optimizations, we evaluated the VC-PWDF score between the initial and final geometries for each candidate structure of each compound. The results are summarized by the box plot in Fig. 3 ▸, where the boxes span the interquartile range of the VC-PWDF values. The whiskers span 95% of the data, while outliers are shown as individual points. For context, a VC-PWDF score of < 0.03 is usually a good indicator of significant structural similarity for rigid molecules and < 0.05 for more flexible molecules (Mayo & Johnson, 2021 ▸; Mayo *et al.*, 2022 ▸).

From Fig. 3 ▸, compound XXXIII shows the largest structural changes during optimization, followed by compound XXVIII, despite both systems comprising fairly rigid molecules. It is reasonable that the force field would be less accurate for the organic salt (XXXIII) due to the importance of electrostatic interactions. Similarly, the organometallic copper complex (XXVIII) may be problematic due to difficulties in parameterization for copper and the unpaired electron in the complex. Instances of large geometric changes with VC-PWDF > 0.1 also occurred for some candidate structures of compounds XXXI and XXXII, although the ranges spanned by 95% of the points are much narrower compared to those for XXVIII and XXXIII.

As it is fairly common for force fields to predict more energy minima than DFT (Price, 2013 ▸; Neumann & van de Streek, 2018 ▸; Francia *et al.*, 2020 ▸; Butler & Day, 2023 ▸), we also assess how many of the candidate structures were duplicates (*i.e.* corresponding to the same polymorph) before and after geometry optimization. This provides insight into whether different force-field local minima correspond to a single DFT minimum. Since we are comparing structures that were all optimized with the same method (*i.e.* either the CSD-tailored force field or DFT), we do not need to consider variable cells and we can use the PWDF scores as our similarity metric. Here, two structures are deemed equivalent if their PWDF value is < 0.01.

The results in Table 2 ▸ show the number of duplicate structures before and after optimization. For the CCDC provided structures, there were only two sets of duplicates each for XXVII (structure IDs 28/61 and 63/77) and XXXII (IDs 58/66 and 169/488). These may have initially been deemed different by COMPACK due to problems with the graph-matching algorithm for highly branched, pseudosymmetric molecules (Mayo *et al.*, 2022 ▸), such as XXVII, or due to the choice of distance and angle tolerances. After optimization, at least two pairs of duplicate structures are seen for all the compounds, with many duplicate structures occurring for XXVIII and XXXIII in particular. This finding is consistent with the larger structural changes seen for these two compounds in Fig. 3 ▸. An example was already discussed in Section 2.2 for compound XXVIII, where two structures (IDs 145 and 207) optimize to become equivalent to the seed structure for the experimental form (ID 144, see Fig. 2 ▸).

### 3.2. BT7-predicted GGA landscapes

**Figure 4:**
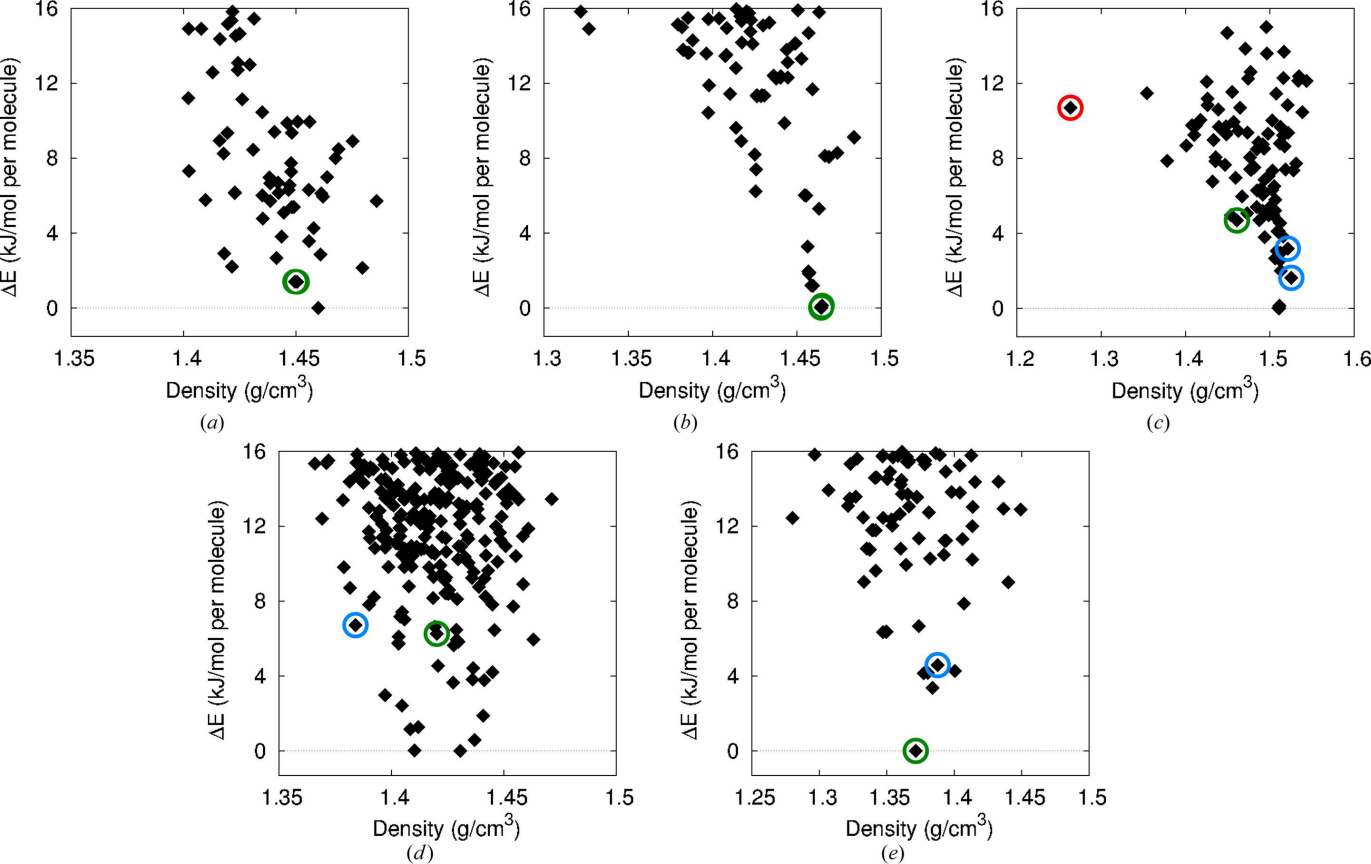
Computed crystal energy landscapes obtained using B86bPBE-XDM with the light basis set: (*a*) XXVII, (*b*) XXVIII, (*c*) XXXI, (*d*) XXXII, (*e*) XXXIII. The green circles indicate the structure that is the best match to the most-stable experimental polymorph, while the blue circles indicate the structure of a second, less-stable polymorph, including major and minor components for compound XXXI. For this same compound, an additional low-density form with large crystal voids, indicated by the red circle, was identified after removal of solvent from a solvate structure.

We consider now the computed crystal energy landscapes obtained from the geometry relaxations with B86bPBE-XDM and the light NAO basis set; these results are shown in Fig. 4 ▸. For compound XXVII, two identical seed structures (IDs 28 and 61), chosen to correspond to the low-temperature experimental form, were included in the set of candidates provided by the CCDC. This seed structure was ranked second lowest on the computed crystal-energy landscape in Fig. 4 ▸(*a*). As it lies < 1.5 kJ mol^−1^ above the global minimum structure (ID 58), which is well within the accepted energy window for the stability of experimentally observable polymorphs (Nyman & Day, 2015 ▸), we consider this to be a successful prediction. Additionally, this energy difference is small enough that thermal free-energy corrections may reverse the ranking (Nyman & Day, 2016 ▸, 2015 ▸; Weatherby *et al.*, 2022 ▸). Rotations of the isopropyl groups, which become disordered at room temperature, can also significantly affect the energy ranking (Hunnisett *et al.*, 2024*a* ▸).

For the copper compound, XXVIII, only a single polymorph was identified experimentally. The crystal-energy landscape in Fig. 4 ▸(*b*) shows that the experimental seed structure was ranked as the most stable candidate, again indicating a successful crystal structure prediction. As noted above, two additional candidates became equivalent to the seed structure after geometry optimization.

For compound XXXI, Fig. 4 ▸(*c*) shows that the DFT calculations predict form C to lie quite high in energy, as expected due to the large voids in this crystal structure that formed upon solvent evaporation. While this form lies 10.7 kJ mol^−1^ above the global minimum, significantly outside the usual ‘polymorph window’ (< 7.2 kJ mol^−1^ above the global minimum in 95% of cases) (Nyman & Day, 2015 ▸), it has been shown that similar high-energy, low-density structures can be isolated from solvates (Yang & Day, 2021 ▸). Turning to the more stable experimental forms, our calculations predict the seed structures for form B and the major/minor components of form A to all lie within 5 kJ mol^−1^ of the global minimum. However, the predicted stability ordering is incorrect, with form B ranked as the least stable, lying 4.7 kJ mol^−1^ above the global minimum and 3.1 kJ mol^−1^ above the major component of form A.

As shown in Fig. 4 ▸(*d*), our calculations were quite unsuccessful in identifying the experimental forms of compound XXXII as being low in energy amongst the candidates. Here, both forms were found to lie 6–7 kJ mol^−1^ in energy above the global minimum. The more stable form B was ranked 22nd in energy, while form A was ranked 26th, recovering the correct experimental ordering, but with a predicted energy difference of only 0.4 kJ mol^−1^ separating the two forms.

Finally, for compound XXXIII, Fig. 4 ▸(*e*) shows that form B is correctly ranked as the most stable of the candidate structures, constituting another successful prediction. Form A is also found to be a fairly stable structure, ranked sixth in energy and lying < 5 kJ mol^−1^ above form A, which is still within the accepted polymorph window (Nyman & Day, 2015 ▸).

### 3.3. Dependence on functional and basis set

In previous benchmarking of XDM-corrected density functionals for the compounds comprising the first six blind tests, we found that hybrid functionals outperformed GGAs in some cases (Price *et al.*, 2023*a* ▸), specifically those with significant delocalization error (Bryenton *et al.*, 2023 ▸). In particular, delocalization error was found to affect the ranking of the organic salts (compounds XIX and XXIV) (Price *et al.*, 2023*a* ▸; Whittleton *et al.*, 2017*a* ▸), as well as for compound X where the intramolecular conformational energy has a substantial contribution to the ranking (Price *et al.*, 2023*a* ▸; Greenwell & Beran, 2020 ▸; Whittleton *et al.*, 2017*b* ▸). Similarly, significant errors in GGA energy rankings have been found for other flexible molecules due to errors in the conformational energy (Greenwell & Beran, 2020 ▸; Greenwell *et al.*, 2020 ▸; Beran *et al.*, 2022 ▸). As a result, we performed single-point energy calculations with the B86bPBE-25X-XDM and B86bPBE-50X-XDM functionals, both with the light basis set, which was expected to lead to more accurate ranking relative to the B86bPBE-XDM results alone. We also performed single-point energy calculations with B86bPBE-XDM and the tight basis set to identify if the errors seen for compounds XXXI and XXXII were a result of basis-set incompleteness. This allowed us to approximate hybrid functional results with the tight basis using an additive scheme (Hoja & Tkatchenko, 2018 ▸; Hoja *et al.*, 2019 ▸; Price *et al.*, 2023*b* ▸): 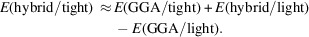


The identities of the minimum-energy structures obtained with each combination of functional and basis set are given in the supporting information. A comparison of the overall rankings and relative energies of the experimental seed structures, relative to the respective global minimums, are shown in Table 3 ▸. Note that the rankings were determined by taking some pairs of structures as equivalent: the experimental matches, structures 28 and 61, for compound XXVII (POWDIFF = 0.0004); the experimental matches, structures 144, 145 and 207, for compound XXVIII (POWDIFF ≤ 0.0005); and the DFT energy minima, structures 17 and 59, for compound XXXI (POWDIFF = 0.0039). Full crystal-energy landscapes are provided in the supporting information.

For the blind test submission, we selected results from the B86bPBE-25X functional for compounds XXVII and XXVIII, and results from B86bPBE-50X for the remaining three compounds, as we expected them to potentially be more susceptible to delocalization error due to being able to adopt a range of stable conformations (XXXI and XXXII) or being an organic salt (XXXIII). However, the results in Table 3 ▸ show that these were not the optimal choices. Overall, all three functionals give very similar ranking for compounds XXVII, XXVIII, and XXXIII. It would seem that the crystal energy landscapes of these three compounds are relatively easy to model accurately with dispersion-corrected DFT, since the ranking does not depend on the choice of functional in any consequential way. This consistence was expected for the first two compounds, as they are relatively rigid, non-polar molecules, and their crystals are therefore unlikely to have much inherent delocalization error.

**Figure 5:**
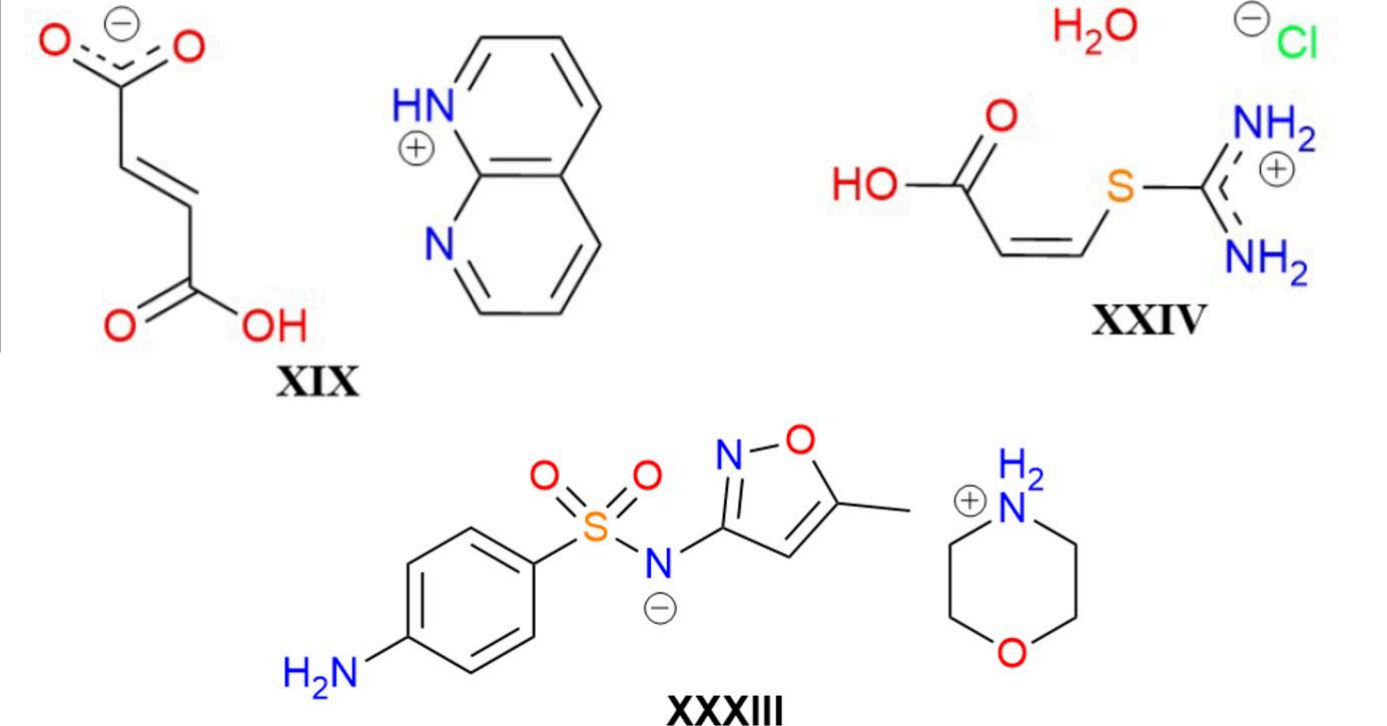
Chemical structures of the three organic salts considered in the CSP blind tests: XIX from BT5, XXIV from BT6, and XXXIII from BT7.

In light of our previous results for the salt compounds XIX and XXIV, the lack of functional dependence was somewhat surprising for compound XXXIII. Therefore, we conclude that not all organic salts are equally affected by delocalization error. One likely explanation for the difference is that delocalization error frequently manifests in cases with extended conjugation (Greenwell & Beran, 2020 ▸; Greenwell *et al.*, 2020 ▸; Beran *et al.*, 2022 ▸; Whittleton *et al.*, 2017*b* ▸) and cooperative hydrogen bonding (Whittleton *et al.*, 2017*a* ▸), such as for COOH groups where the OH can act as a hydrogen-bond donor and the =O can act as a hydrogen-bond acceptor. As seen from their chemical structures, shown in Fig. 5 ▸, moieties in salts XIX and XXIV both exhibit extended conjugation, with all species being planar, and one ion in each possesses COOH groups that form cooperative hydrogen-bonds in the crystal (Whittleton *et al.*, 2017*a* ▸; Price *et al.*, 2023*a* ▸). Conversely, for XXXIII, the cation is not planar, the conjugation in the anion is broken by the SO_2_ group, and neither can participate in cooperative hydrogen bonding.

We now move to the two flexible molecules, compounds XXXI and XXXII. In sharp contrast to the other three members of this blind test, these compounds proved extremely challenging for DFT and also exhibit more functional dependence. From Table 3 ▸, the experimental forms of compounds XXXI and XXXII are ranked increasingly higher in energy (with respect to the global minimum) with increasing fractions of exact exchange. Increasing exact exchange does lower the energy of form B relative to the major component of form A for compound XXXI, but not enough to recover the correct ordering. However, for compound XXXII, form B was correctly predicted to be more stable than the major component of form A with the GGA functional and this ordering is reversed with both hybrids. Therefore, our choice of 50% exact exchange for the blind test submission was unfortunate here and this demonstrates how difficult it can be to know, a priori, whether delocalization error will affect a CSP landscape. Finally, we find that the tight basis set does not improve the rankings, widening the energy gap between forms A and B for compound XXXI and raising the experimental forms of compound XXXII to substantially higher energies. This allows us to rule out basis-set incompleteness for the very electronegative F atoms appearing in these two compounds as the source of the poor rankings.

### 3.4. Vibrational free-energy corrections

To summarize our results so far, the DFT energy rankings were quite successful for three of the blind test compounds (XXVII, XXVIII and XXXIII), but performed much less well for the landscapes of XXXI and XXXII. Notably, these are both fairly large, flexible molecules with several rotatable bonds. This is reminiscent of compound XX from the fifth CSP blind test, another large, flexible molecule for which our DFT energy rankings showed considerable errors (Price *et al.*, 2023*a* ▸). We previously conjectured that thermal free-energy corrections, neglected in zeroth-order CSP (Price, 2018 ▸), may be responsible for the poor energy ranking here, and this may also be the case for compounds XXXI and XXXII. To test this hypothesis, phonon calculations were performed for the 16 lowest-energy candidates for compound XXXI and the 26 lowest-energy candidates for compound XXXII at the B86bPBE-XDM/light level. These ranges of structures were selected to include all of the experimental seed structures, except for the high-energy solvate (see Table 3 ▸).

**Figure 6:**
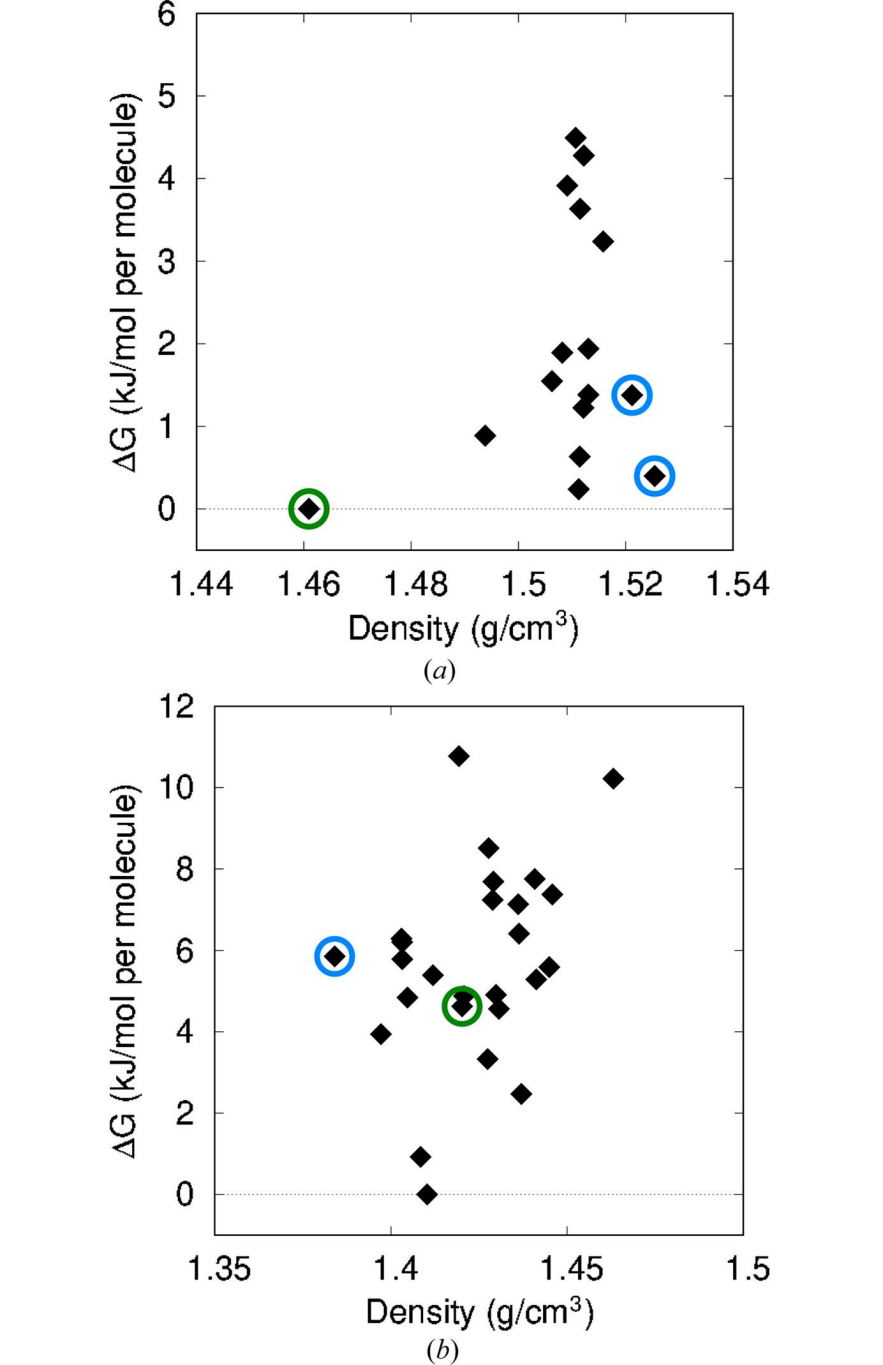
Computed crystal free-energy landscapes for a temperature of 298 K obtained using B86bPBE-XDM with the light basis set: (*a*) XXXI and (*b*) XXXII. Phonon calculations to evaluate the thermal free-energy corrections were only performed for the 16 lowest-energy structures of compound XXXI and the 26 lowest-energy structures of compound XXXII. The green circles indicate the structure that is the best match to the most-stable experimental polymorph, while the blue circles indicate the structure of a second, less-stable polymorph, including major and minor components for compound XXXI.

**Figure 7:**
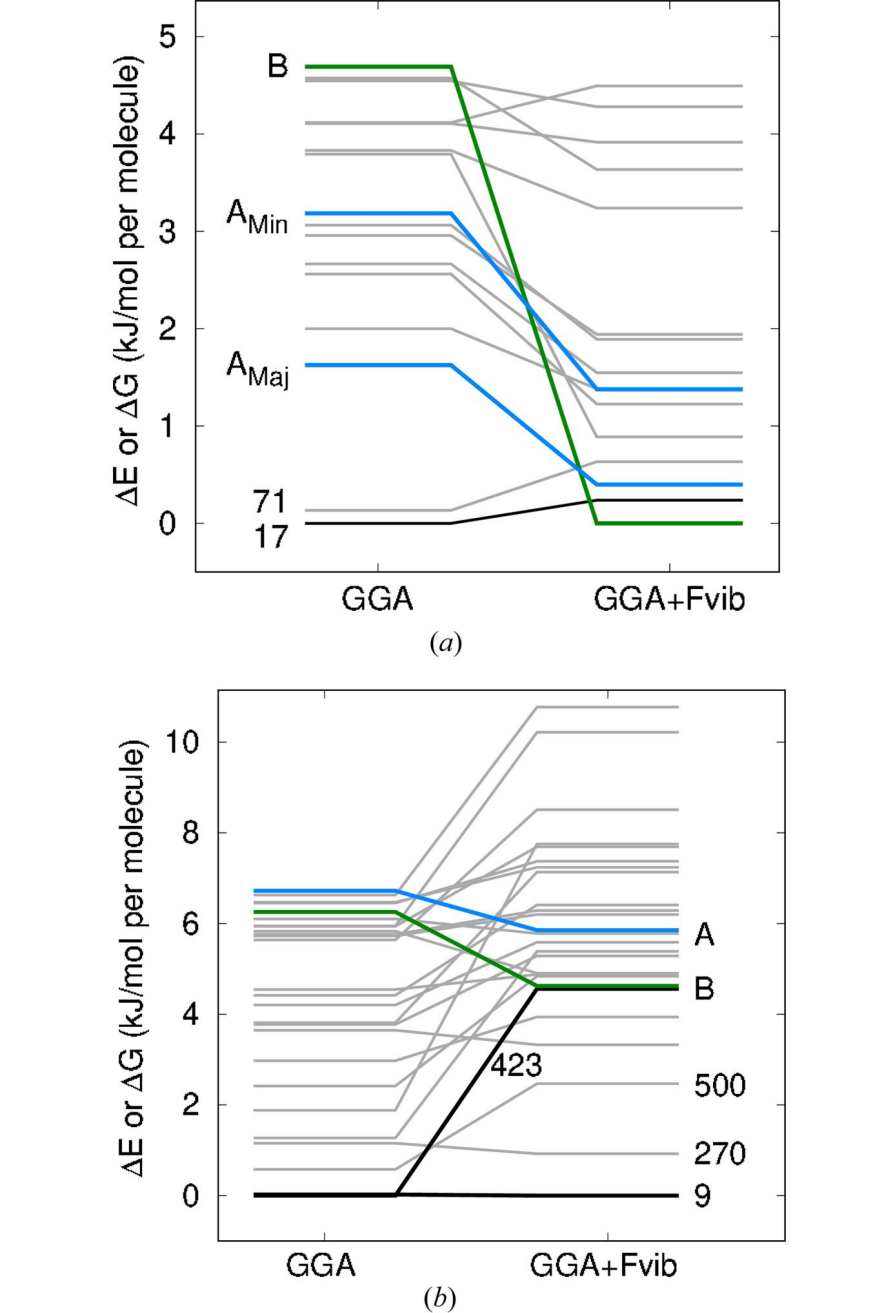
Changes in polymorph ranking with B86bPBE-XDM/light upon inclusion of thermal free-energy energy corrections: (*a*) XXXI and (*b*) XXXII. The GGA results correspond to relative electronic energies, while GGA+Fvib corresponds to relative free energies for a temperature of 298 K.

Fig. 6 ▸ shows the free-energy landscapes for compounds XXXI and XXXII, where the thermal free-energy corrections for a temperature of 298 K have been added to the B86bPBE-XDM/light electronic energies. Free-energy landscapes obtained using different base functionals and/or basis sets for the electronic energies are shown in the supporting information. Fig. 7 ▸ shows the change in ranking of the considered structures upon inclusion of the thermal free-energy corrections.

The results in Figs. 6 ▸(*a*) and 7 ▸(*a*) show that inclusion of thermal free-energy terms corrects the energy ranking for compound XXXI. Using the electronic energies alone, form B was found to lie 4.7 kJ mol^−1^ above the DFT minimum. However, using free energies, form B is successfully predicted to be the global minimum structure, while the major component of form A is ranked third, <0.5 kJ mol^−1^ above form B. Experimentally, form B is the more stable form up to a temperature of 48–55°C (Hunnisett *et al.*, 2024*b* ▸), after which the disordered form A is more stable. It should be noted, however, that this ranking only applies to the subset of structures for which free-energy calculations were performed. It is possible that some structures with even higher lattice energies would also be stabilized sufficiently by inclusion of free-energy corrections to affect these rankings.

Figs. 6 ▸(*b*) and 7 ▸(*b*) show that inclusion of thermal free-energy corrections also improves the ranking for compound XXXII. Here, form B is now ranked seventh with a Δ*G* of 4.6 kJ mol^−1^, while form A is ranked fifth with a Δ*G* of 5.9 kJ mol^−1^, in contrast to the rankings of 22nd and 26th in Table 3 ▸ obtained with electronic energies alone. However, the two experimental forms do remain fairly high in free energy above the global minimum. This finding suggests that (i) there are errors in our DFT treatment, (ii) anharmonicity and/or thermal expansion is important in determining the vibrational free-energy correction and the quasi-harmonic approximation is needed, (iii) kinetic and/or surface-energy effects prevent formation of the lower-energy candidates identified with DFT, or (iv) some combination of these factors (Price, 2013 ▸).

## 4. Conclusions

In this work, we applied XDM-corrected DFT methods to the energy ranking phase of the seventh CSP blind test. Large changes in some of the provided candidate structures were observed after DFT geometry optimization, particularly for compounds XXVIII and XXXIII. Additionally, many candidates collapsed to the same local minimum, giving duplicate structures after optimization. This points to the importance of developing improved force fields for the initial structure generation and geometry optimization steps of CSP. In terms of the DFT energy ranking itself, our methodology gave successful predictions of the most stable experimentally isolated polymorphs as being the lowest or second-lowest energy structures for compounds XXVII, XXVIII and XXXIII. However, the most stable experimental polymorphs were ranked relatively high in energy for compounds XXXI and XXXII.

We conclude that our DFT methodology provides good reliability for CSP studies of fairly rigid molecules, but there remain challenges for large and highly flexible pharmaceutical targets. We suggest that thermal free-energy corrections may be much more important for such structures than previously demonstrated for smaller, more rigid molecules (Nyman & Day, 2016 ▸; Nyman & Day, 2015 ▸; Weatherby *et al.*, 2022 ▸). While the thermal free-energy corrections are typically ≤ 2 kJ mol^−1^, they amounted to as much as 4.7 and 5.9 kJ mol^−1^ for compounds XXXI and XXXII, respectively. Previous work found even larger thermal free-energy corrections, up to 8.4 kJ mol^−1^, for compound XXIII in the sixth blind test (Hoja & Tkatchenko, 2018 ▸; Hoja *et al.*, 2019 ▸). Here, the most stable isolated polymorph for compound XXXI was successfully predicted as the global minimum with DFT upon inclusion of a vibrational free-energy correction. This correction improved the ranking for compound XXXII as well, although the experimental structures were still ranked sixth and 15th in energy. It is possible that corrections beyond the harmonic approximation are required, or that kinetic and/or surface energy effects prevent crystallization of the lower-energy candidates identified with DFT in this case.

## Supplementary Material

Energy rankings. DOI: 10.1107/S2052520624002774/aw5086sup1.txt

Tables I, II, III, and 19 extra figures. DOI: 10.1107/S2052520624002774/aw5086sup2.pdf

## Figures and Tables

**Table 1:** Structure numbers for the experimental seed structures provided by the CCDC Also shown are three metrics to compare the provided and DFT-optimized crystal structures; these are the RMSD(20) obtained from the COMPACK algorithm (Motherwell & Chisholm, 2005 ▸) implemented in *Mercury* (Macrae *et al.*, 2020 ▸), as well as the powder difference (PWDF) and variable-cell powder difference (VC-PWDF) (Mayo *et al.*, 2022 ▸) evaluated using *critic2* (Otero-de-la-Roza *et al.*, 2014 ▸).

Compound	Form	Number	RMSD(20) (Å)	PWDF	VC-PWDF
XXVII	LT	28 = 61	0.483	0.049	0.004
XXVIII	–	144†	0.098	0.031	0.004
XXXI	A_Maj_	98	0.124	0.042	0.004
	A_Min_	1	0.241	0.098	0.012
	B	25	0.174	0.038	0.005
	C	89	0.106	0.032	0.004
XXXII	A_Maj_	317	0.137	0.023	0.002
	B_RT_	232	0.265	0.036	0.003
XXXIII	A	233	0.114	0.033	0.005
	B	452	0.159	0.061	0.006

† 145 and 207 were also equivalent to this structure after geometry optimization.

**Table 2:** Number of duplicate structures for each compound, as quantified by a PWDF score of 0.01 or less between pairs of candidates Results are shown for both the initial structures provided by the CCDC and those after DFT geometry optimization.

	No. of duplicates	
Compound	CCDC	DFT
XXVII	2	2
XXVIII	0	40
XXXI	0	4
XXXII	2	8
XXXIII	0	27

**Table 3:** Summary of computational results For each compound, the energy rank of all isolated polymorphs and their relative energies (Δ*E* in kJ mol^−1^ per molecule, or per molecule pair in the case of the salt) above the minimum on the corresponding crystal energy landscape are shown for each of the methods used.

		Light						Tight					
		GGA		25X		50X		GGA		25X		50X	
Compound	Form	Rank	Δ*E*	Rank	Δ*E*	Rank	Δ*E*	Rank	Δ*E*	Rank	Δ*E*	Rank	Δ*E*
XXVII	LT	2	1.4	2	1.0	2	1.0	3	1.4	2	1.2	3	1.0
XXVIII	–	1	0.0	1	0.0	1	0.0	1	0.0	1	0.0	1	0.0
XXXI	A_Maj_	3	1.6	5	2.7	7	3.9	3	0.5	3	1.6	7	2.8
	A_Min_	9	3.2	10	4.4	15	5.8	4	1.6	8	2.9	9	4.2
	B	16	4.7	16	5.3	13	5.7	21	4.6	18	5.3	15	5.6
	C	77	10.7	73	11.5	66	11.9	80	10.2	76	10.9	66	12.2
XXXII	A_Maj_	26	6.7	24	8.8	30	11.2	34	9.6	31	10.2	32	10.8
	B_RT_	22	6.3	28	9.3	41	12.6	20	8.6	30	10.2	39	11.6
XXXIII	A	6	4.6	5	4.5	5	5.2	6	5.7	5	5.6	6	6.4
	B	1	0.0	1	0.0	1	0.0	1	0.0	1	0.0	1	0.0
