# Supplementary material for: Assessment of the exchange-hole dipole moment dispersion correction for the energy ranking stage of the seventh crystal structure prediction blind test
Source: Acta Crystallogr B Struct Sci Cryst Eng Mater. 2024 Oct 15;80(Pt 6):595–605. doi: 10.1107/S2052520624002774 (PMC11789164; doi:10.1107/S2052520624002774)
Supplement: Supplementary file 2 [file b-80-00595-sup2.pdf]

STRUCTURAL SCIENCE
CRYSTAL ENGINEERING
MATERIALS

Volume 80 (2024)

Supporting information for article:

Assessment of the exchange-hole dipole moment dispersion correction for the energy ranking stage of the seventh crystal structure prediction blind test

R. Alex Mayo, Alastair J. A. Price, Alberto Otero-de-la-Roza and Erin R. Johnson

Assessment of XDM-corrected density functionals for the energy ranking stage of the 7th CSP blind test

R. Alex Mayo,[1] Alastair J. A. Price,[1] Alberto Otero-de-la-Roza,[2, a)] and Erin R. Johnson[1, b)]

[1)] *Department of Chemistry, Dalhousie University, 6243 Alumni Crescent, Halifax, Nova Scotia, B3H 4R2, Canada*

[2)] *Departamento de Química Física y Analítica and MALTA-Consolider Team, Facultad de Química, Universidad de Oviedo, 33006 Oviedo, Spain*

(Dated: 18 March 2024)

TABLE I. Total computation times in core years for the GGA geometry optimizations and subsequent single-point energy evaluations. Most calculations used 40 cores 2.5 GHz Intel Xeon Gold 6248 processors. The total time is 469.55 core years.

Compound	Opt	25X	50X	Tight
XXVII	58.52	0.82	0.90	1.65
XXVIII	187.60	8.27	12.74	15.23
XXXI	5.77	0.15	0.17	0.81
XXXII	106.84	1.98	2.04	3.37
XXXIII	58.95	1.02	1.04	1.68
Total	417.68	12.24	16.89	22.74

TABLE II. Identity of the minimum energy structure obtained with each level of theory. Also listed are the minimum free-energy structures obtained for compounds XXXI and XXXII only.

Compound	Light			Tight		
	GGA	25X	50X	GGA	25X	50X
XXVII	58	58	58	58	58	58
XXVIII	144=145=207			144=145=207		
XXXI	17=59	17=59	17=59	17=59	17=59	17=59
XXXII	423	500	500	423	423	423
XXXIII	452	452	452	452	452	452
Including thermal free-energy corrections						
XXXI	25	17=59	17=59	98	17=59	17=59
XXXII	9	270	500	270	270	500

TABLE III. Supercell sizes used in phonon calculations for compounds XXXI and XXXII.

XXXI		XXXII	
Candidate	Supercell	Candidate	Supercell
1	3×2×1	7	3×1×1
11	3×3×1	9	2×2×2
17	4×1×1	57	2×2×1
20	2×2×2	82	2×2×2
23	3×2×1	114	3×1×1
25	3×2×1	117	2×2×1
28	3×2×1	130	2×2×1
30	3×2×1	140	2×2×1
32	4×2×1	165	2×2×1
34	3×3×1	193	3×2×1
57	3×2×1	199	3×1×1
63	3×3×2	232	2×2×1
70	3×2×1	236	2×2×1
71	4×1×1	250	3×2×1
73	4×2×1	270	2×2×1
98	3×2×1	299	3×2×1
99	3×3×1	317	2×2×1
		320	2×2×1
		331	2×2×1
		334	2×2×1
		342	2×2×2
		388	2×2×1
		423	2×2×1
		448	2×2×1
		478	2×1×2
		500	3×2×1

[a)] Electronic mail: aoterodelaroza@gmail.com

[b)] Electronic mail: erin.johnson@dal.ca

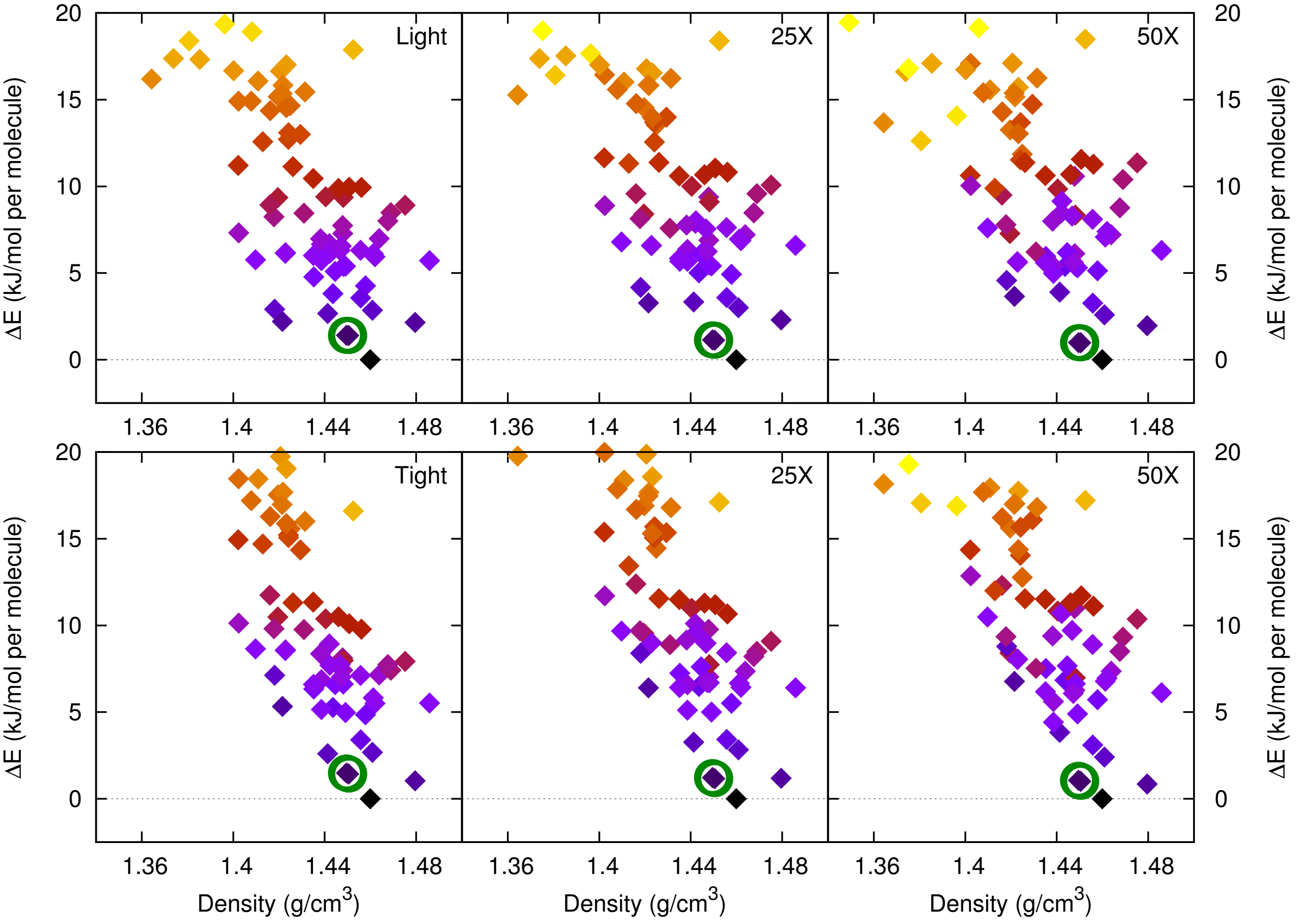

FIG. 1. Computed crystal energy landscapes for compound XXVII. The green circles indicate the structure that is the best match to the most-stable experimental polymorph.

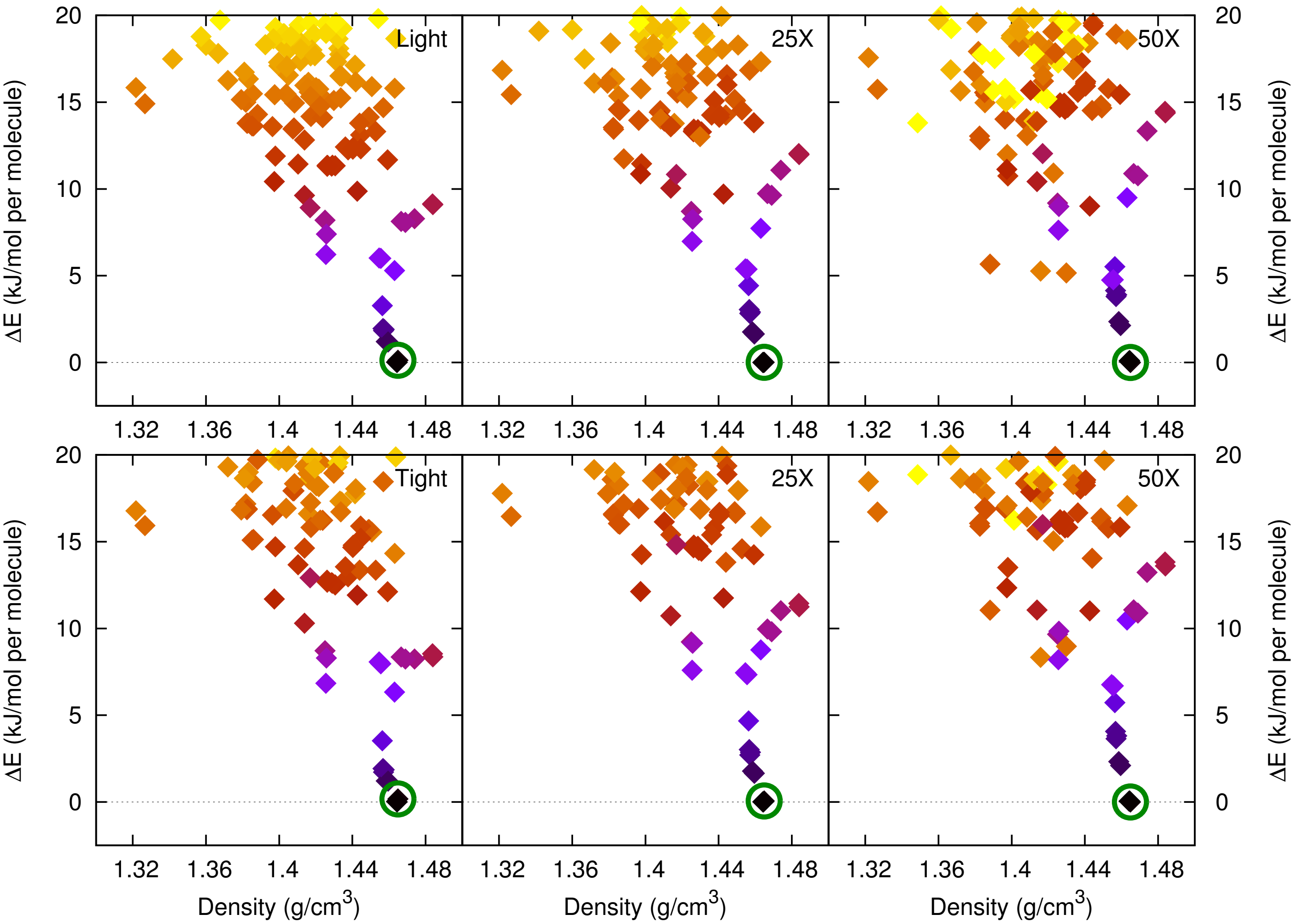

FIG. 2. Computed crystal energy landscapes for compound XXVIII. The green circles indicate the structure that is the best match to the most-stable experimental polymorph.

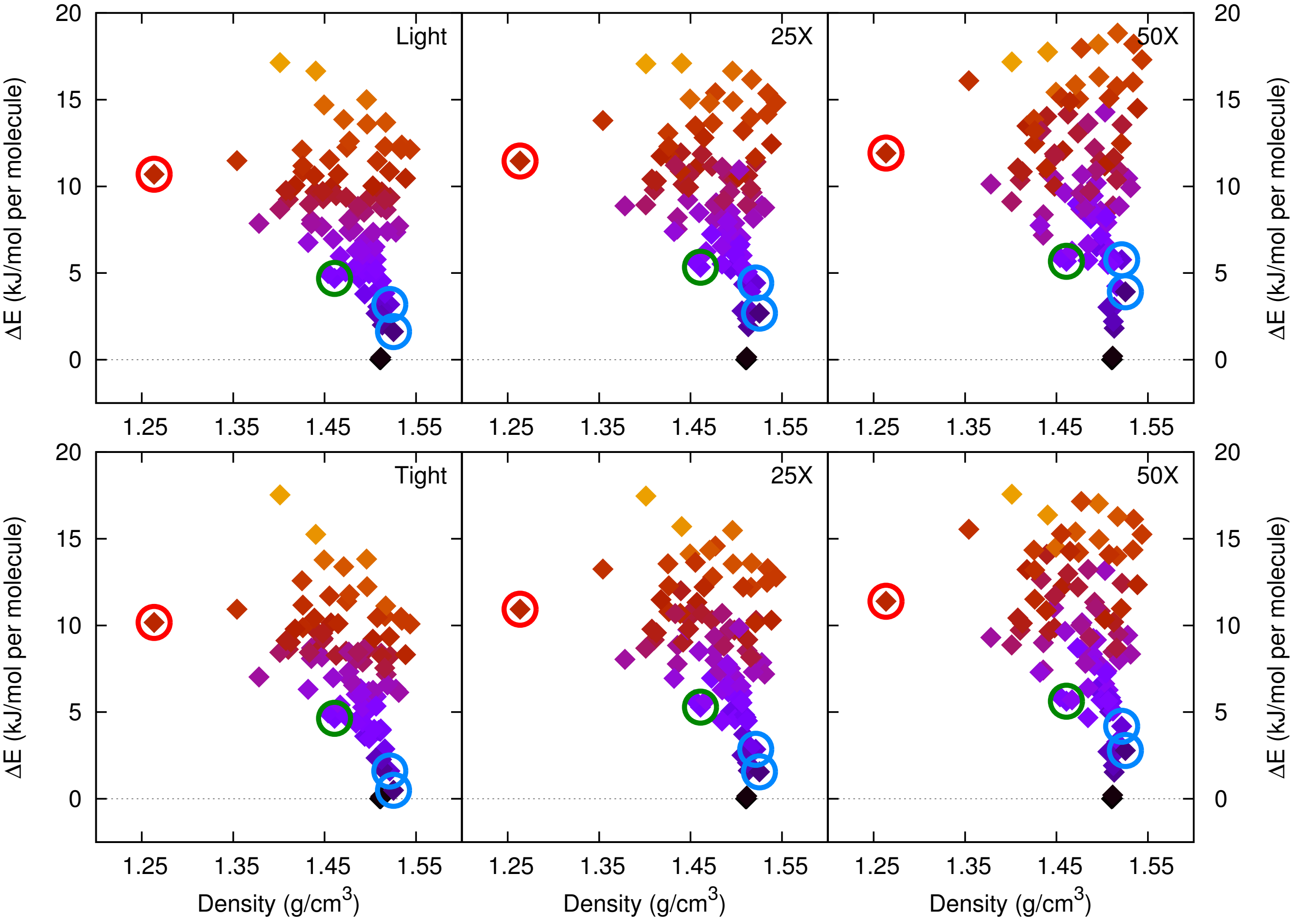

FIG. 3. Computed crystal energy landscapes for compound XXXI. The green circles indicate the structure that is the best match to the most-stable experimental polymorph, while the blue circles indicate the major and minor components of a second, less-stable polymorph. An additional low-density form with large crystal voids, indicated by the red circle, was identified after removal of solvent from a solvate structure.

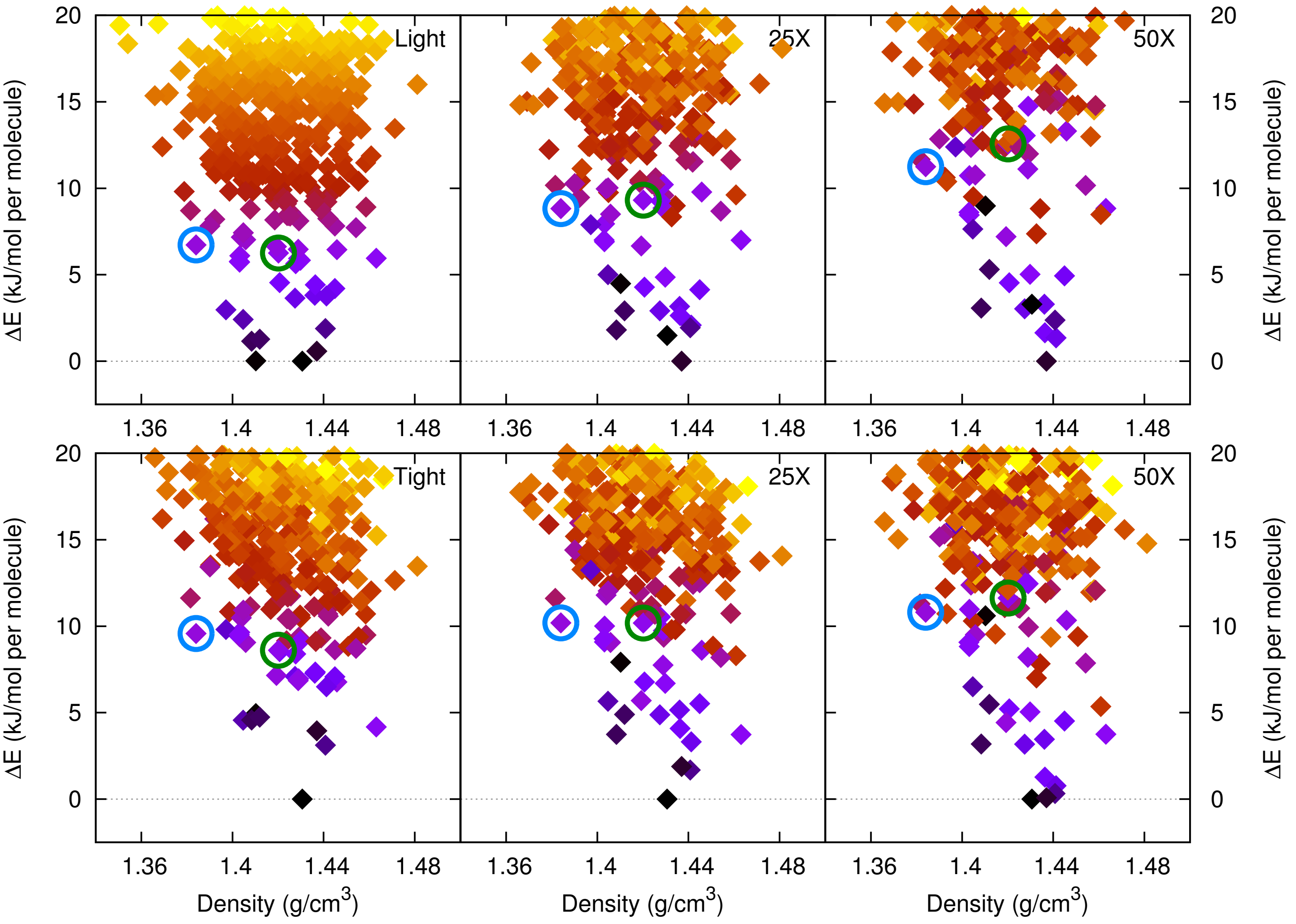

FIG. 4. Computed crystal energy landscapes for compound XXXII. The green circles indicate the structure that is the best match to the most-stable experimental polymorph , while the blue circles indicate the structure of a second, less-stable polymorph.

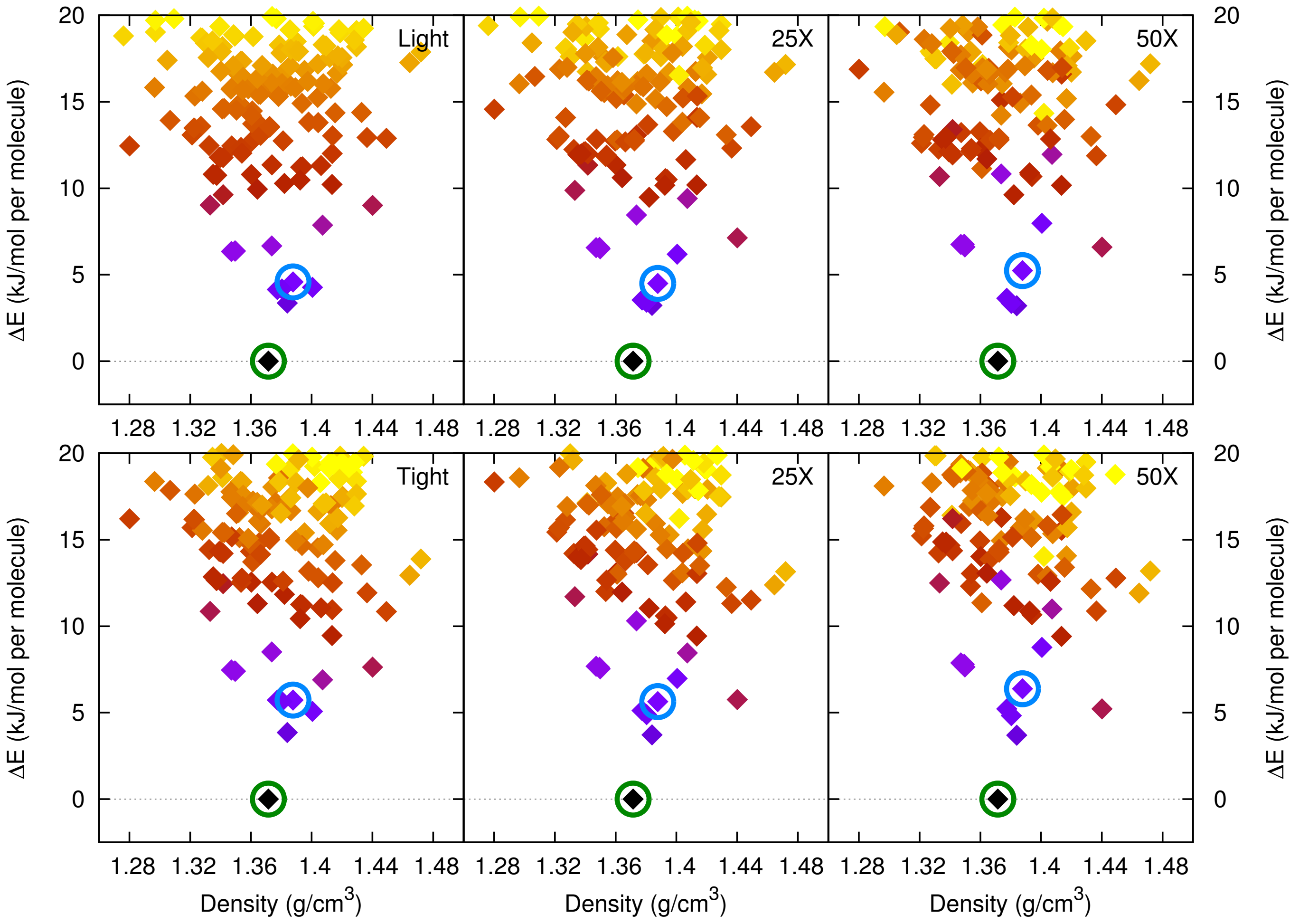

FIG. 5. Computed crystal energy landscapes for compound XXXIII. The green circles indicate the structure that is the best match to the most-stable experimental polymorph , while the blue circles indicate the structure of a second, less-stable polymorph.

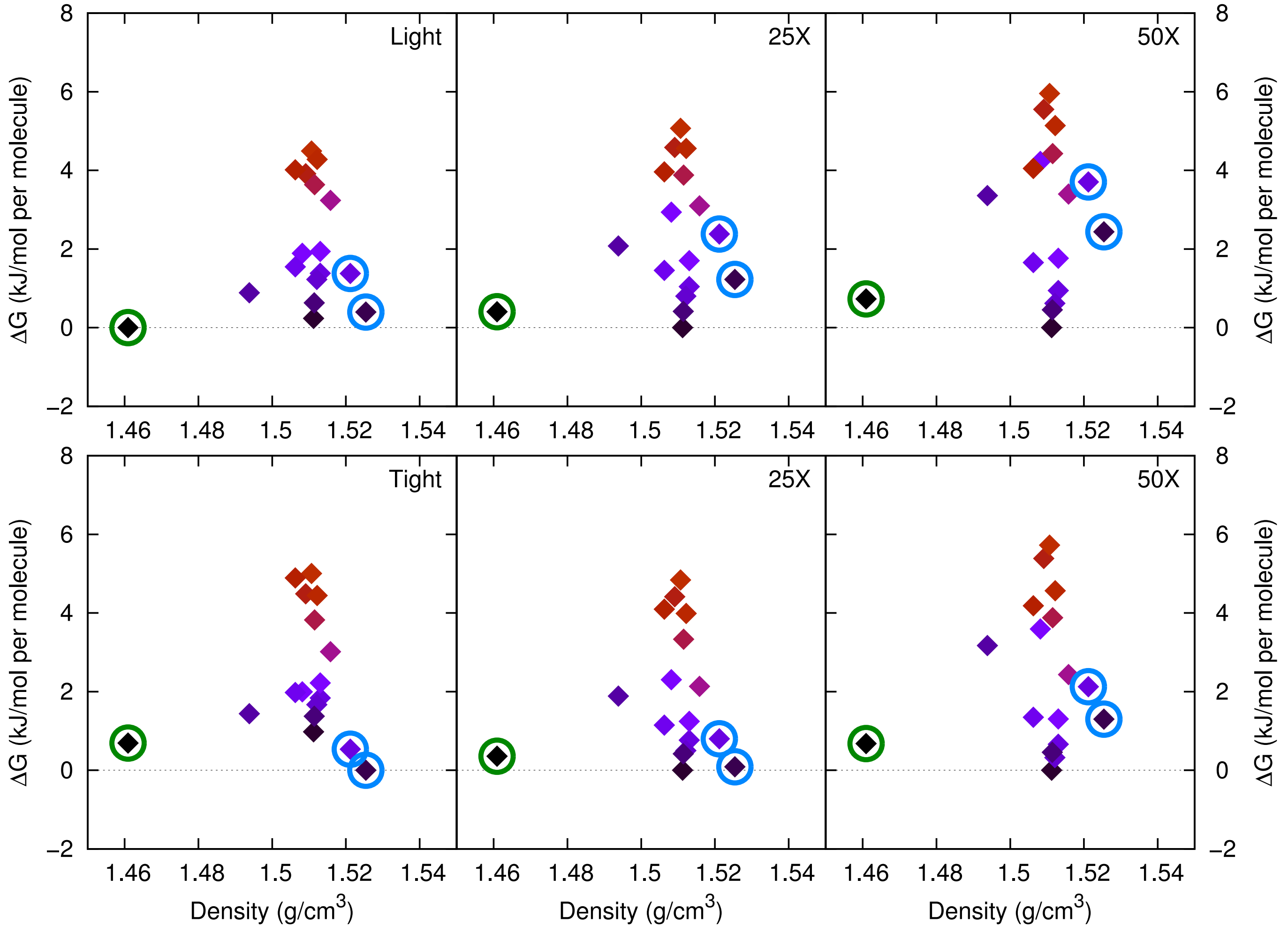

FIG. 6. Computed crystal free-energy landscapes for compound XXXI. The green circles indicate the structure that is the best match to the most-stable experimental polymorph, while the blue circles indicate the major and minor components of a second, less-stable polymorph. Thermal free energy corrections were evaluation from phonon calculations using GGA/Light.

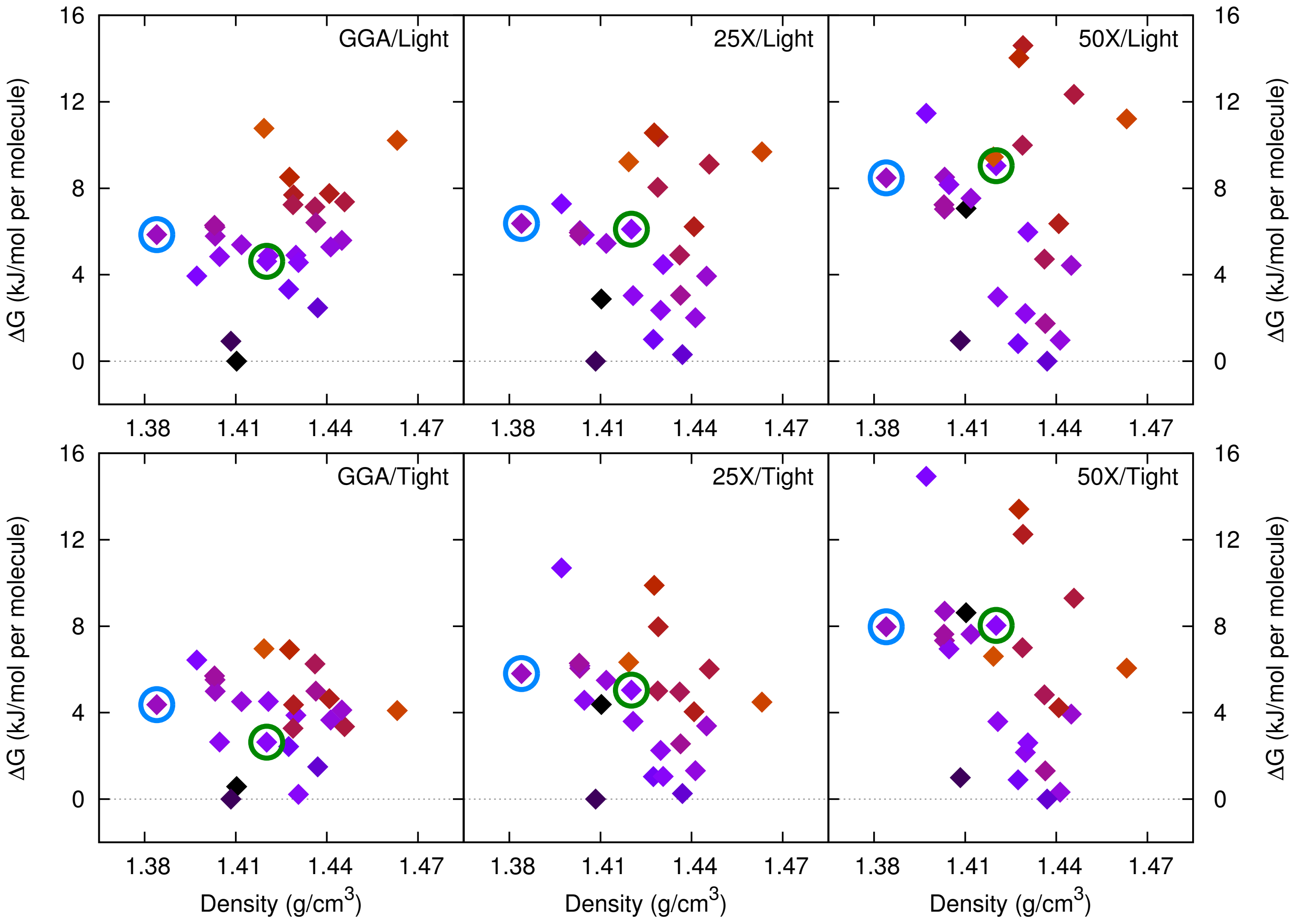

FIG. 7. Computed crystal free-energy landscapes for compound XXXII. The green circles indicate the structure that is the best match to the most-stable experimental polymorph , while the blue circles indicate the structure of a second, less-stable polymorph. Thermal free energy corrections were evaluation from phonon calculations using GGA/Light.

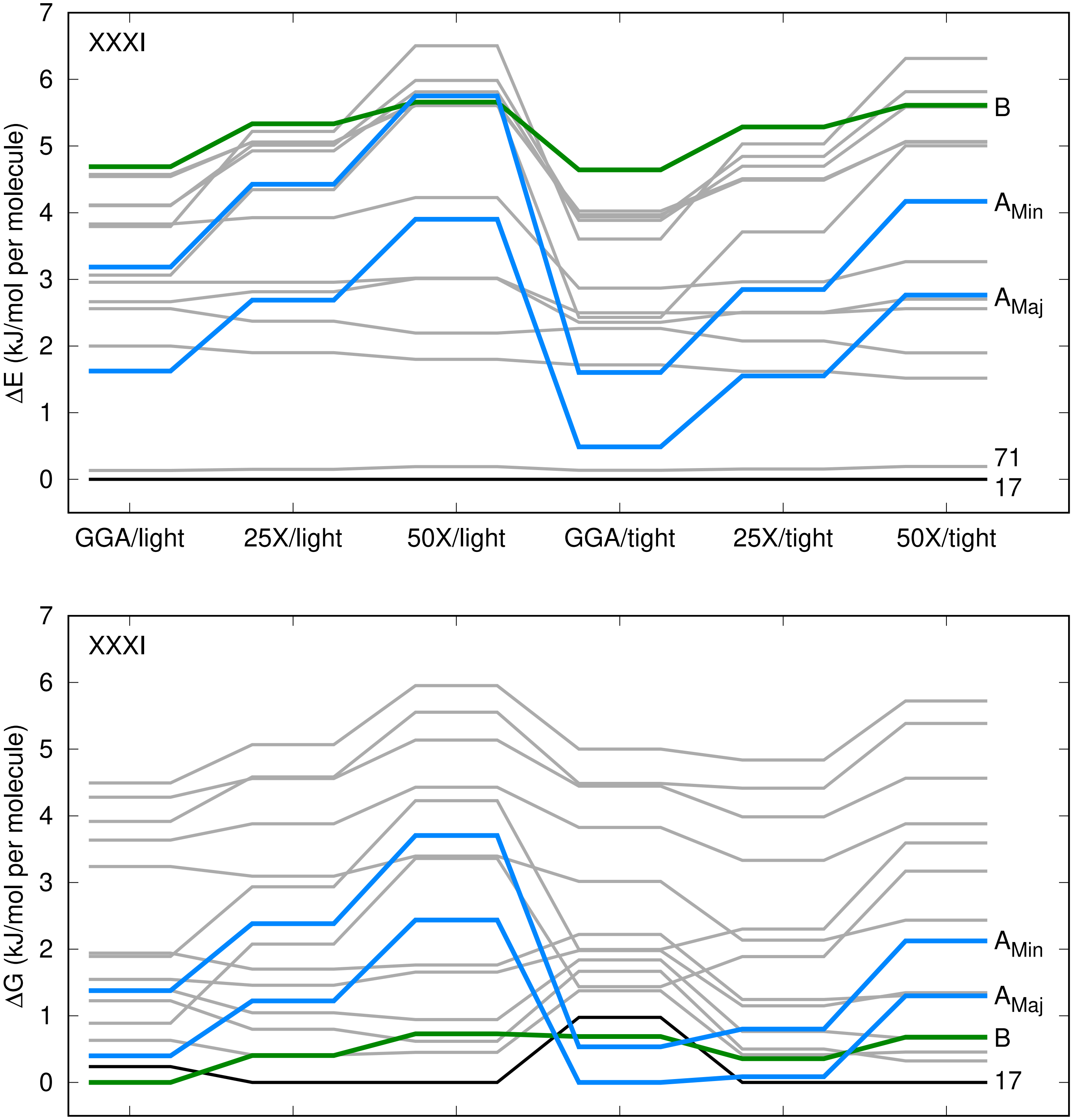

FIG. 8. Changes in polymorph ranking for compound XXXI depending on choice of functional and basis for both relative electronic energies (top) and free energies (bottom).

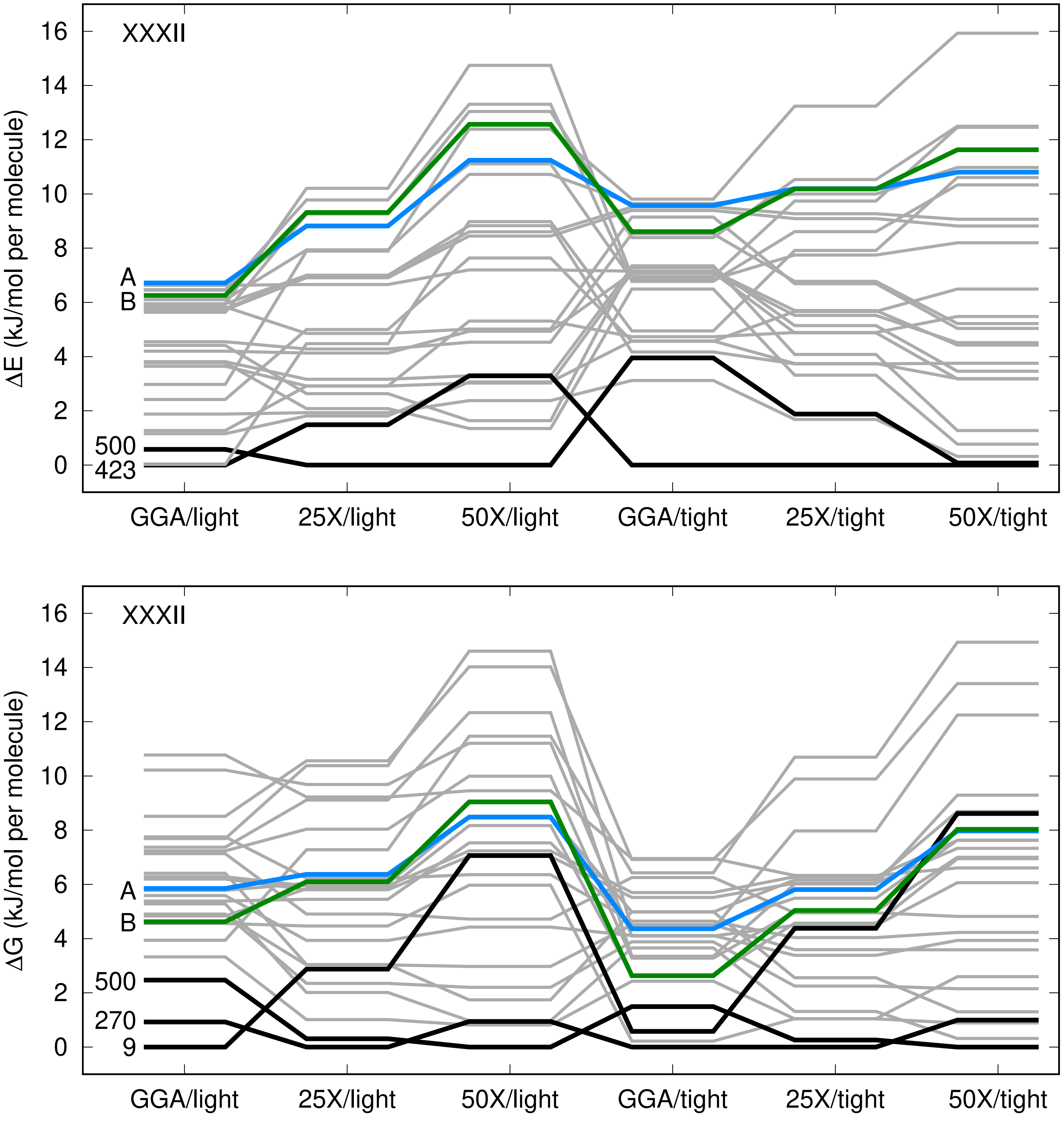

FIG. 9. Changes in polymorph ranking for compound XXXII depending on choice of functional and basis for both relative electronic energies (top) and free energies (bottom).

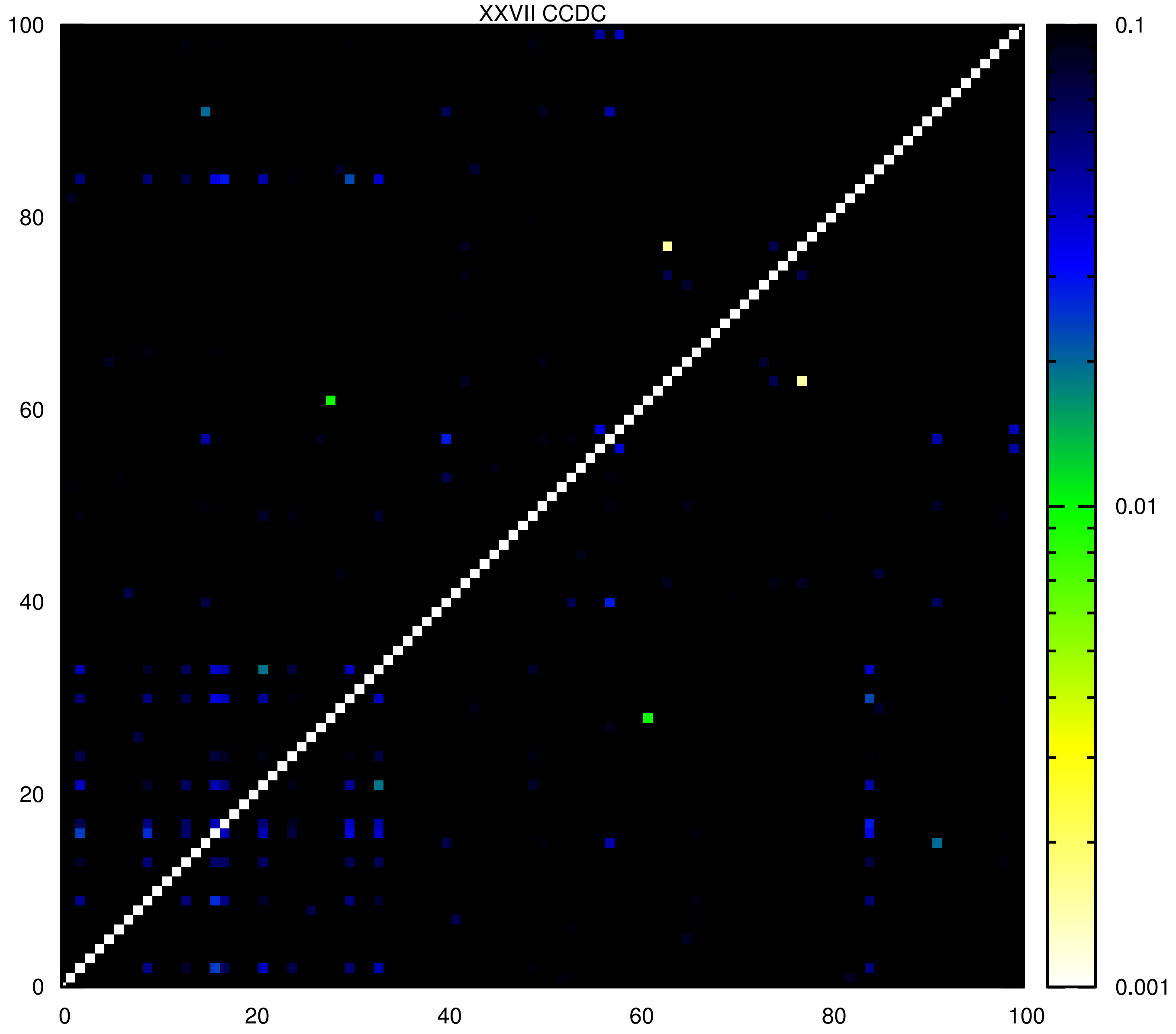

FIG. 10. Heatmap of the PWDF values for comparison of all candidate structures for compound XXVII, using the structures provided by the CCDC.

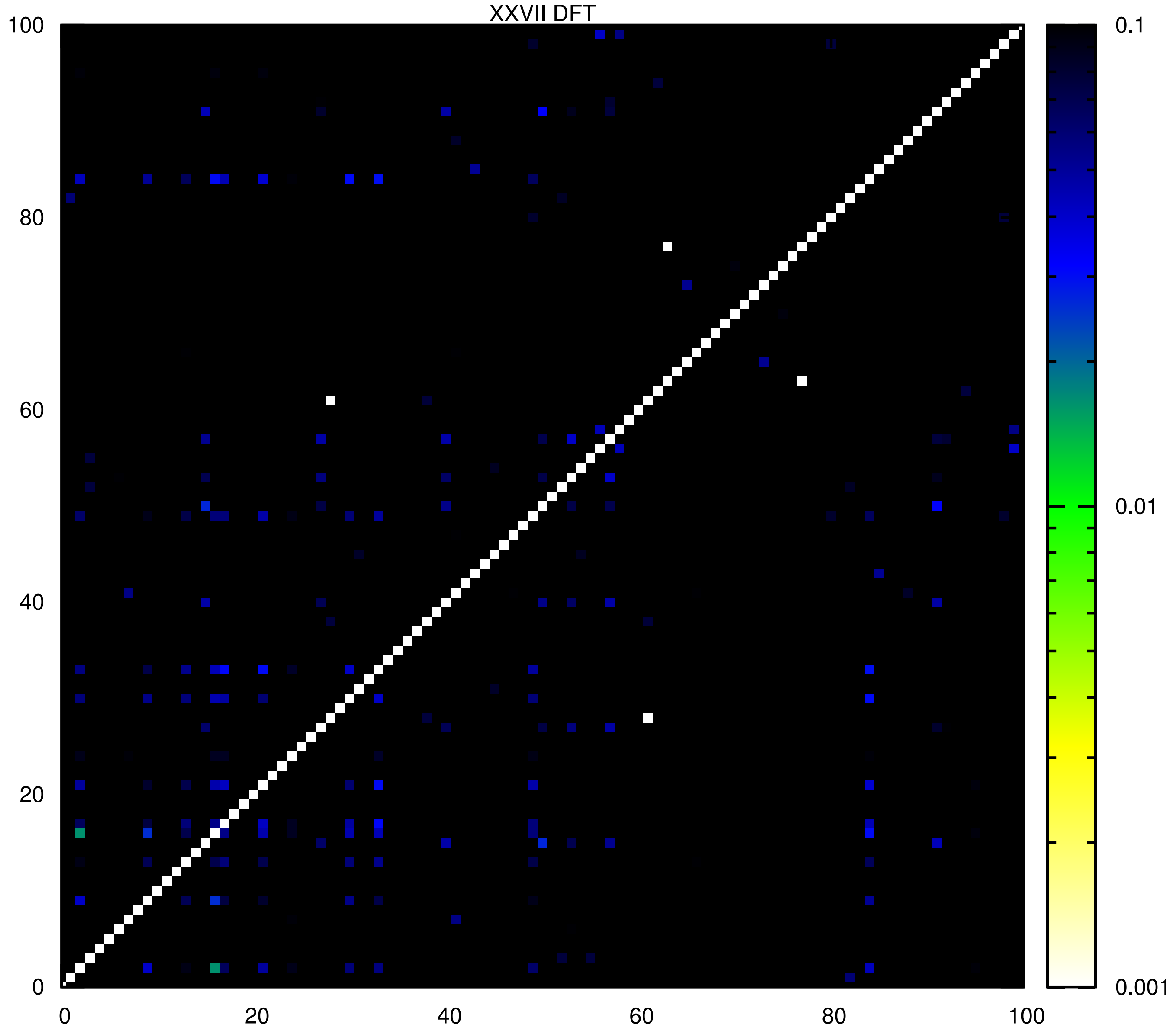

FIG. 11. Heatmap of the PWDF values for comparison of all candidate structures for compound XXVII after DFT geometry optimization.

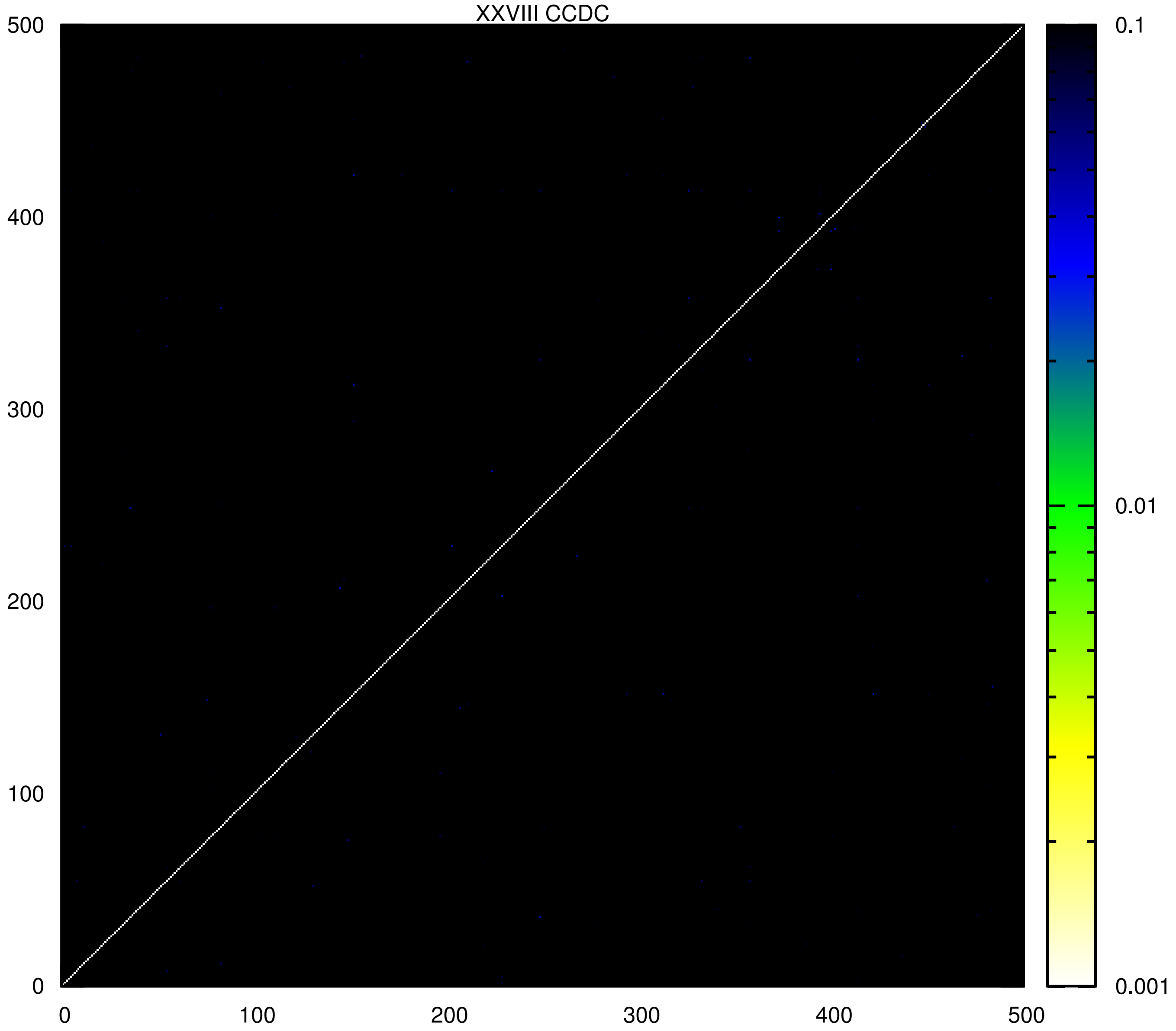

FIG. 12. Heatmap of the PWDF values for comparison of all candidate structures for compound XXVIII, using the structures provided by the CCDC.

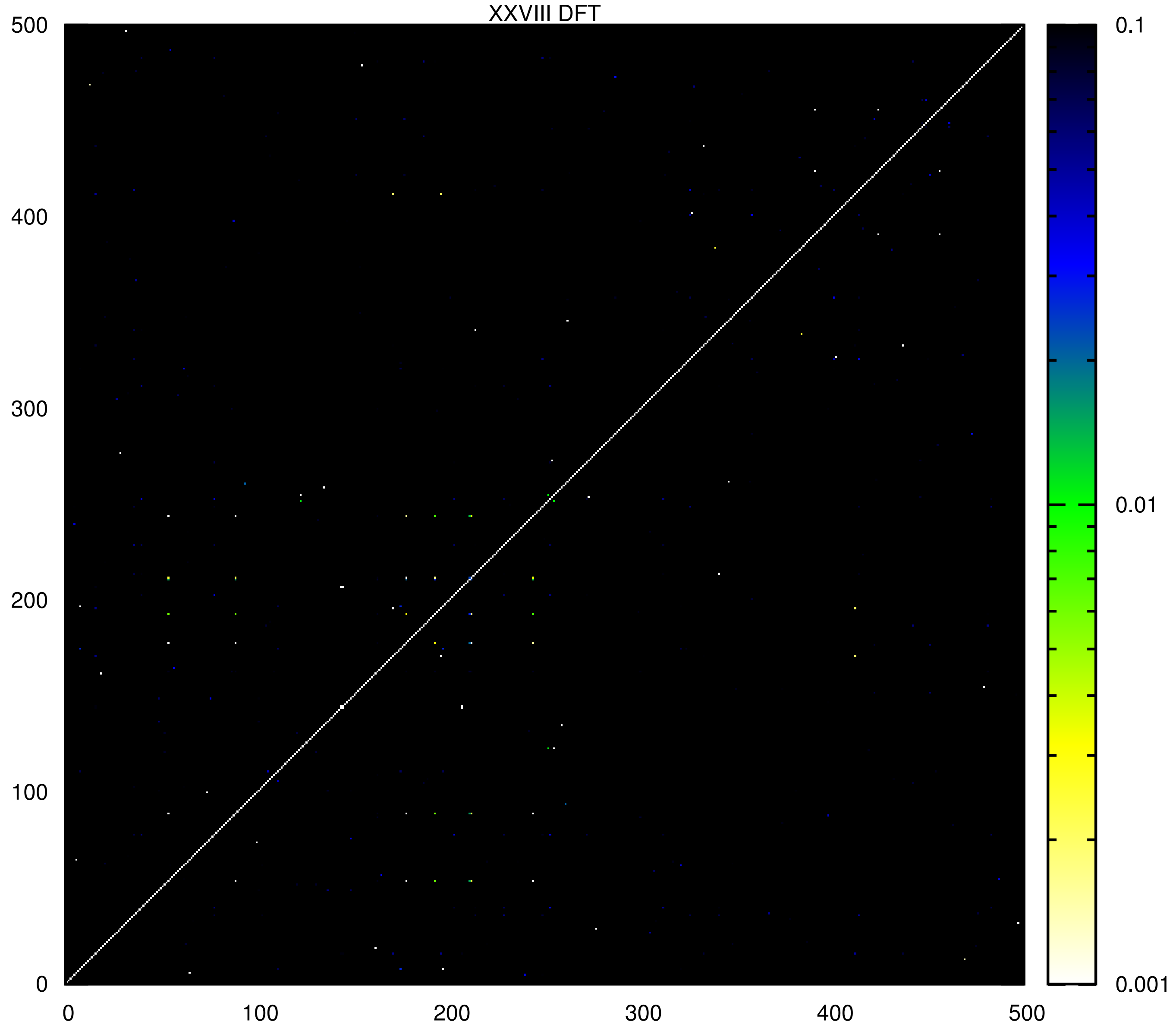

FIG. 13. Heatmap of the PWDF values for comparison of all candidate structures for compound XXVIII after DFT geometry optimization.

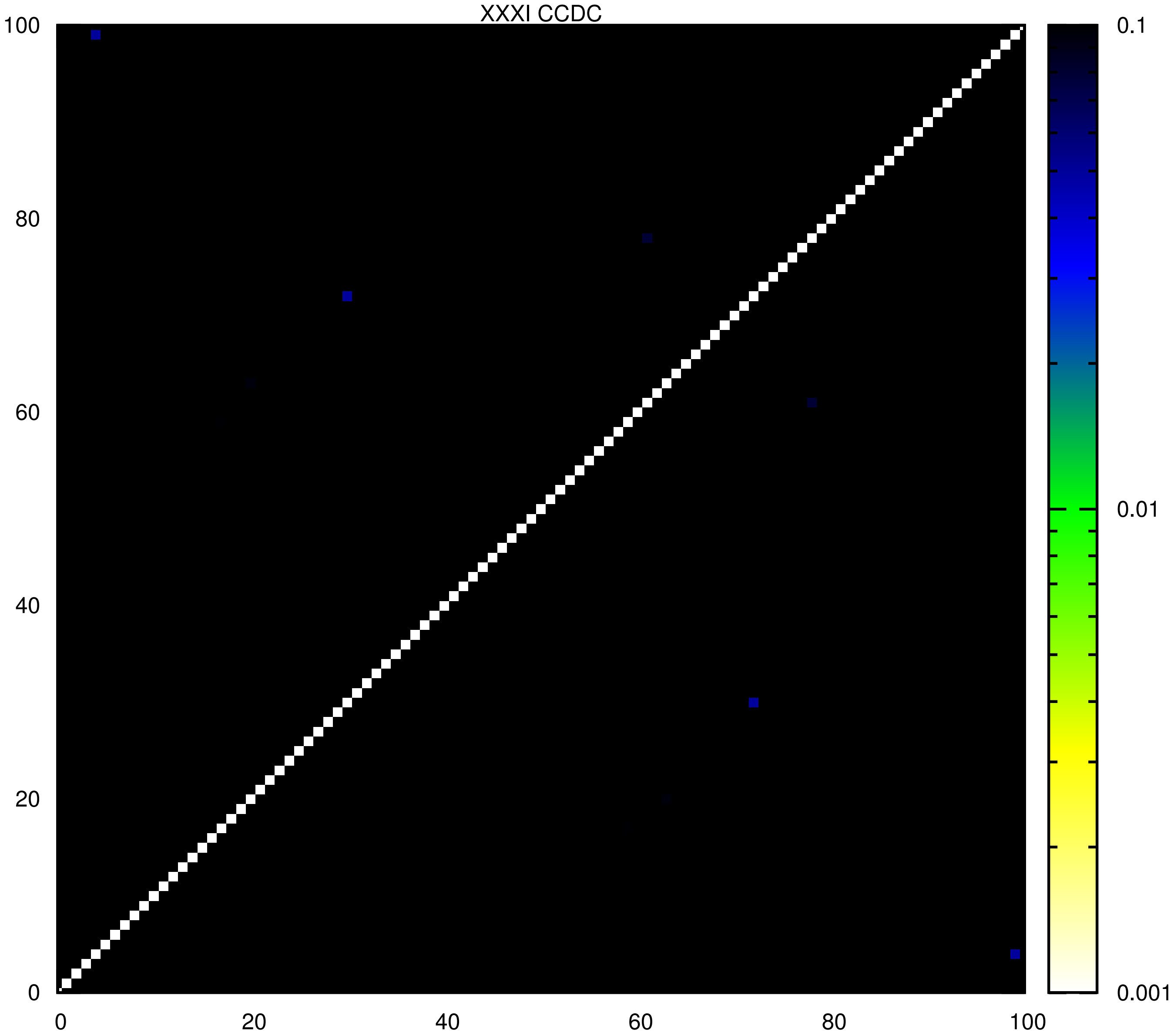

FIG. 14. Heatmap of the PWDF values for comparison of all candidate structures for compound XXXI, using the structures provided by the CCDC.

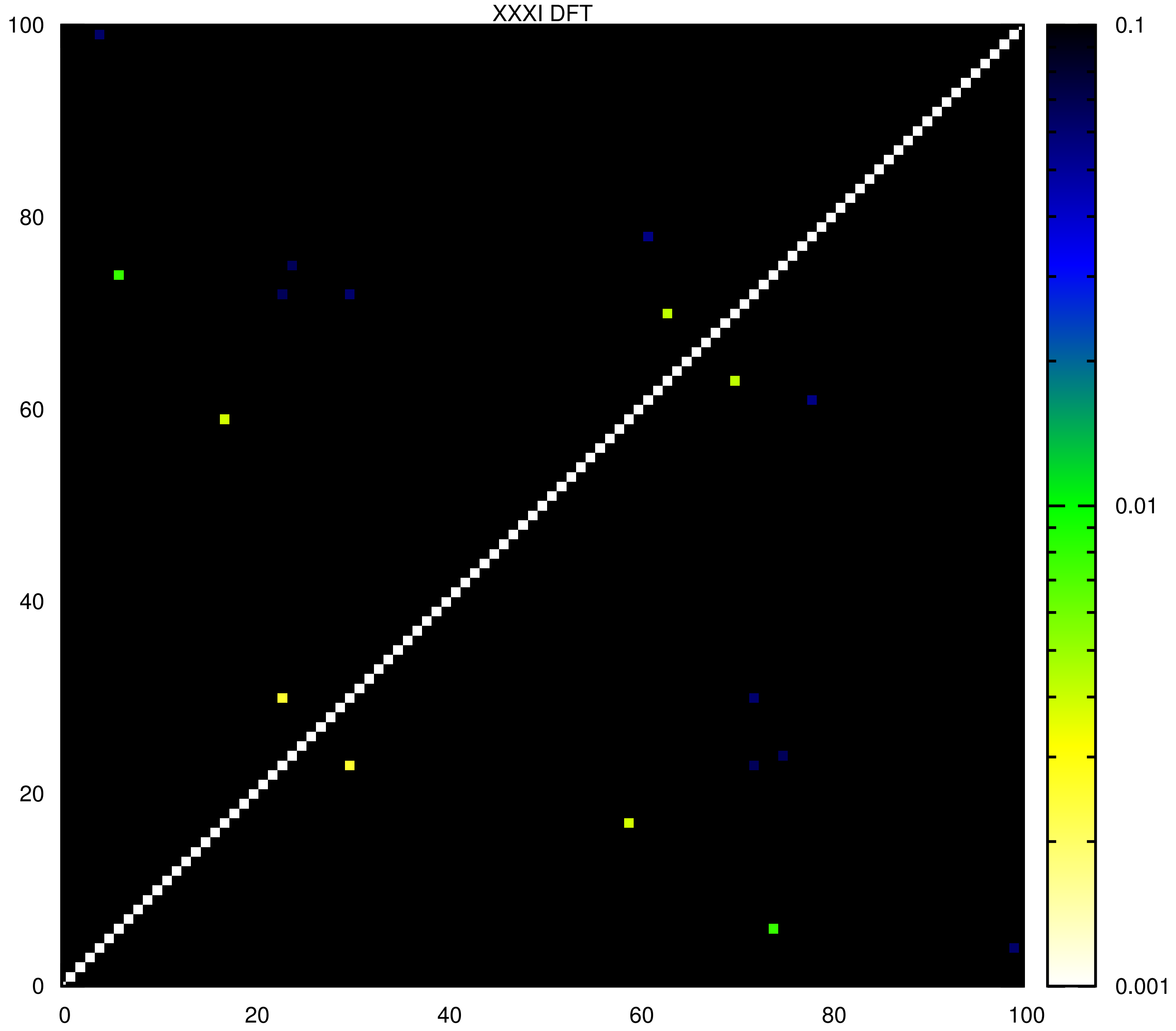

FIG. 15. Heatmap of the PWDF values for comparison of all candidate structures for compound XXXI after DFT geometry optimization.

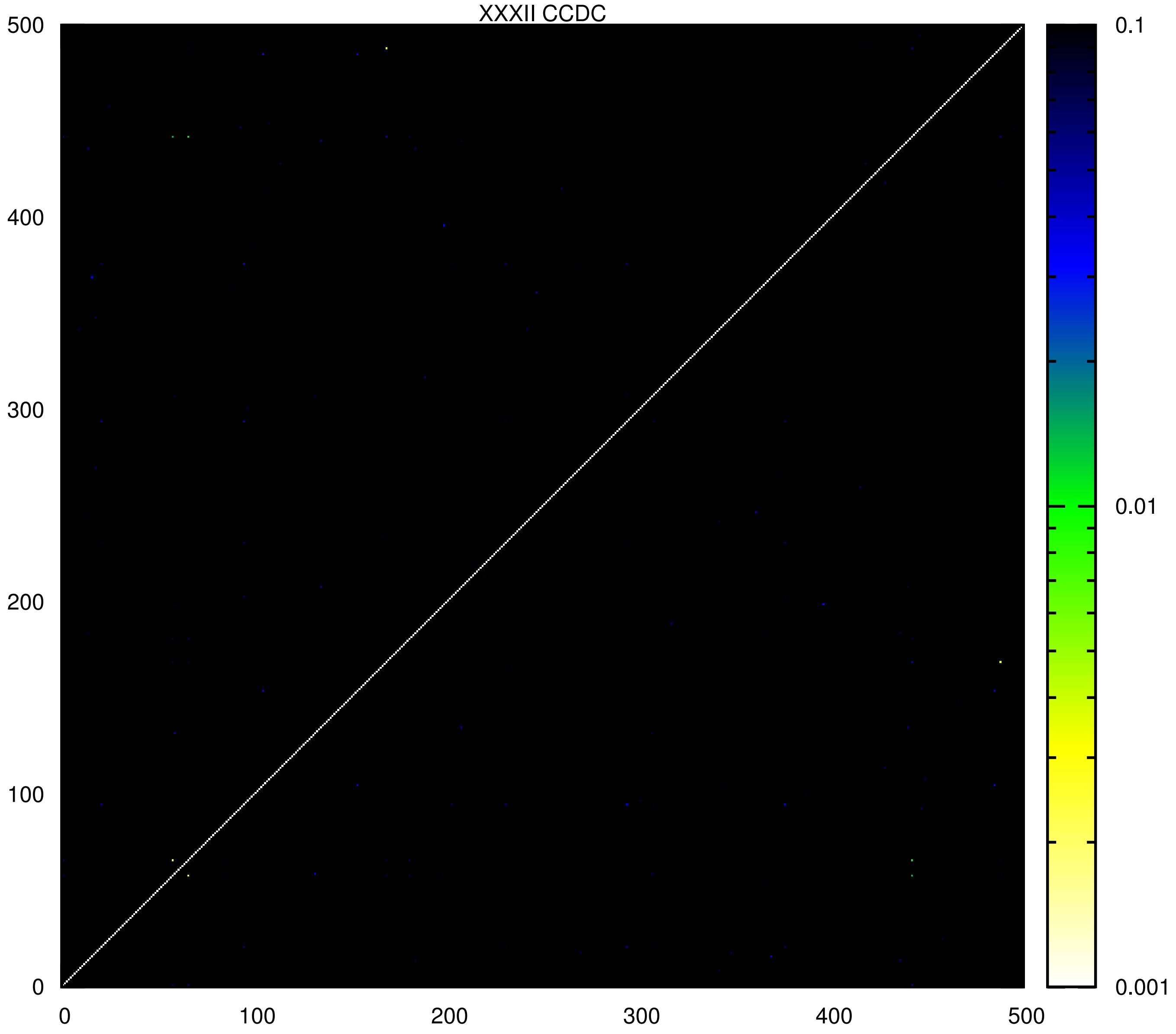

FIG. 16. Heatmap of the PWDF values for comparison of all candidate structures for compound XXXII, using the structures provided by the CCDC.

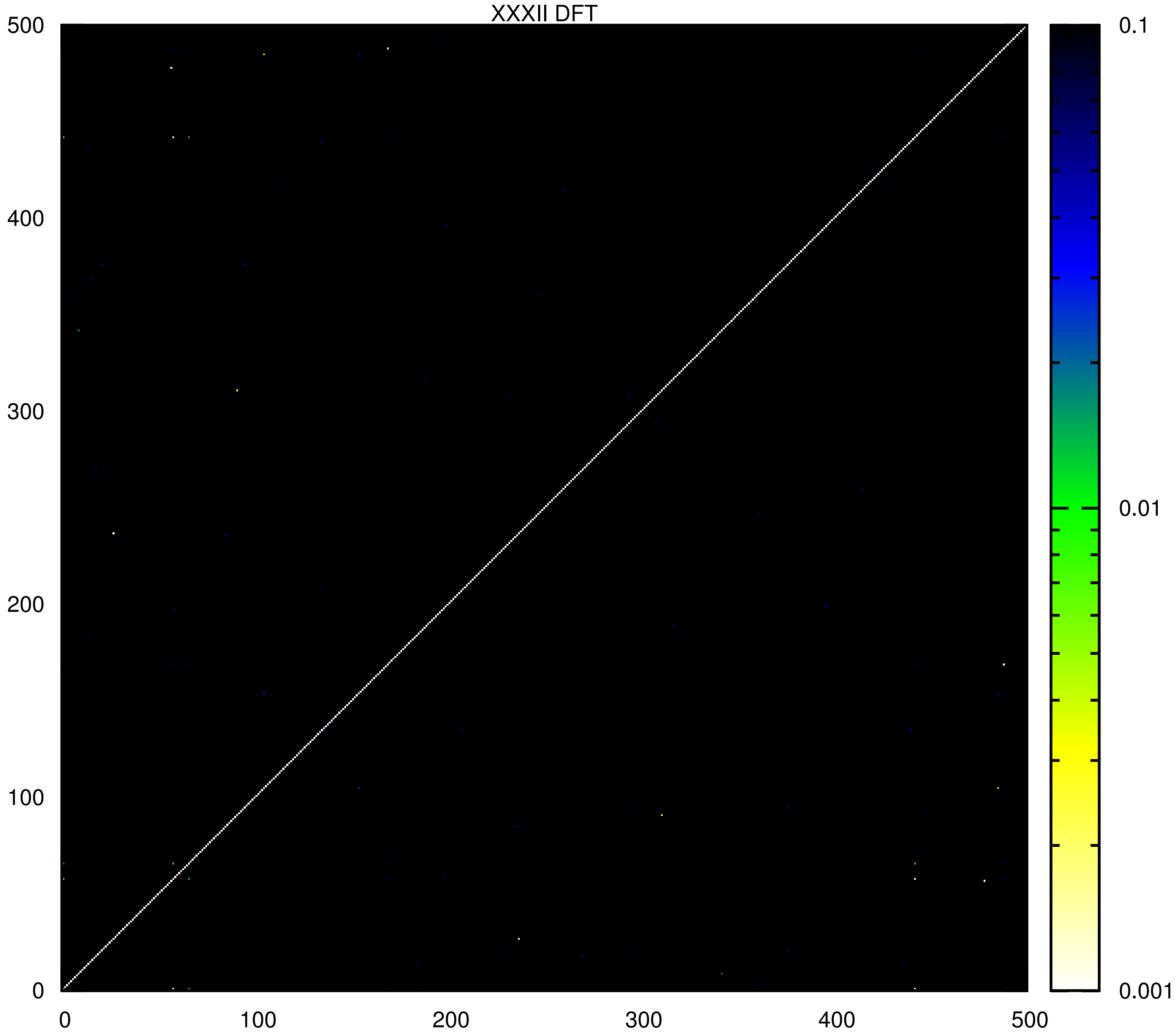

FIG. 17. Heatmap of the PWDF values for comparison of all candidate structures for compound XXXII after DFT geometry optimization.

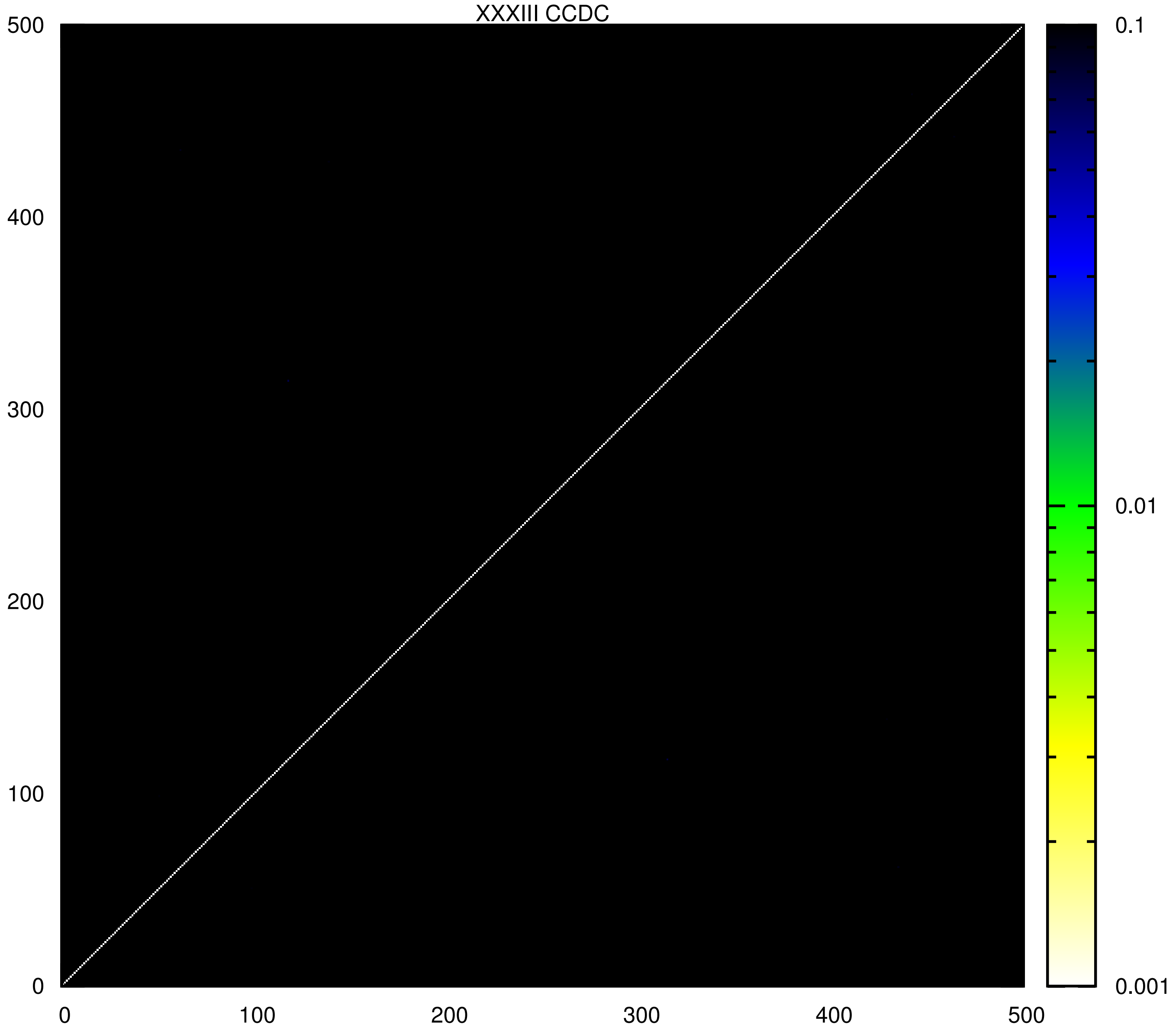

FIG. 18. Heatmap of the PWDF values for comparison of all candidate structures for compound XXXIII, using the structures provided by the CCDC.

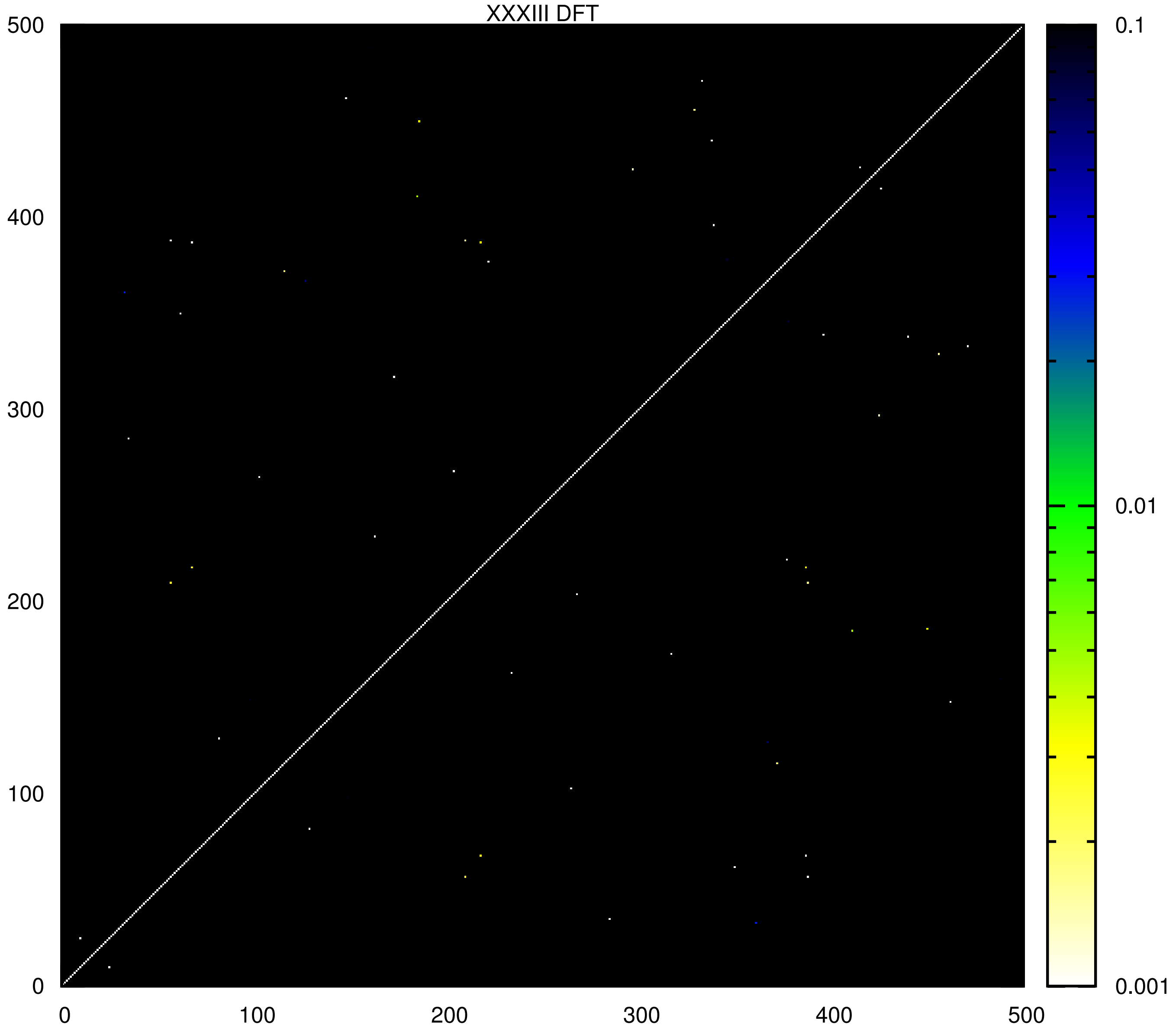

FIG. 19. Heatmap of the PWDF values for comparison of all candidate structures for compound XXXIII after DFT geometry optimization.
